# Altered fecal microbiota composition in individuals who abuse methamphetamine

**DOI:** 10.1038/s41598-021-97548-1

**Published:** 2021-09-13

**Authors:** Yongde Yang, Xuan Yu, Xuebing Liu, Guangya Liu, Kuan Zeng, Gang Wang

**Affiliations:** 1 Wuhan Mental Health Center, The Ninth Clinical School, Tongji Medical College, Huazhong University of Science and Technology, Wuhan, 430030 China; 2 Department of Pathophysiology, School of Basic Medicine, Key Laboratory of Education Ministry of China for Neurological Disorders, Tongji Medical College, Huazhong University of Science and Technology, Wuhan, 430030 China; 3 Department of Applied Psychology, School of Marxism, China University of Geosciences Wuhan, Wuhan, China; 4 Wuhan Jin-yintan Hospital, Wuhan, China

**Keywords:** Microbiology, Diseases

## Abstract

As a severe public health problem, methamphetamine (METH) abuse places a heavy burden on families and society. A growing amount of evidence has indicated communication between gut microbiota and the CNS in drug addiction, with associations to neural, endocrine and immune pathways. Thus, we searched for alterations in the gut microbiota and their potential effects in METH users through 16S rRNA gene sequencing. A decreased Shannon index indicated lower bacterial diversity in the METH users than in the age-matched control group. The gut microbial community composition in the METH users was also altered, including reductions in *Deltaproteobacteria* and *Bacteroidaceae* abundances and increases in *Sphingomonadales*, *Xanthomonadales*, *Romboutsia* and *Lachnospiraceae* abundances. Moreover, the *Fusobacteria* abundance was correlated with the duration of METH use. *Enterobacteriaceae*, *Ruminococcaceae*, *Bacteroides*, and *Faecalibacterium* had statistically significant correlations with items related to the positive and negative symptoms of schizophrenia and to general psychopathology in the METH users, and all have previously been reported to be altered in individuals with psychotic syndromes, especially depression. Abstraction, one of the items of the cognitive assessment, was positively related to *Blautia*. These findings revealed alterations in the gut microbiota of METH users, and these alterations may play a role in psychotic syndrome and cognitive impairment. Although the mechanisms behind the links between these disorders and METH abuse are unknown, the relationships may indicate similarities in the pathogenesis of psychosis induced by METH abuse and other causes, providing a new paradigm for addiction and METH use disorder treatment.

## Introduction

Methamphetamine (METH) abuse has been a severe public health concern in recent decades^1,2^. METH is the most popular addictive drug in China, and there were 118.6 million individuals with METH use disorder in 2019, as reported by the Office of the National Narcotics Control Commission. The release of the neurotransmitter dopamine and decrease in dopamine transporters caused by METH might be the main factors responsible for dose escalation^3^. Chronic METH use causes a selective pattern of cerebral deterioration, including cerebral metabolic abnormalities and structural brain alterations^4^. A meta-analysis showed that impairments of many cognitive domains, such as attention, executive functions, visual memory and working memory, occurred in individuals with METH use disorders^5^. Anxiety or depression, violent behavior (often risk-taking), insomnia, repetitive movements (stereotypy), and psychosis have also been observed with chronic METH use^6^. Individuals with methamphetamine-related disorders also have a higher risk of schizophrenia^7^. However, the mechanisms underlying the cognitive impairment and behavioral abnormalities are still unknown.

Studies of the microbiota–gut–brain axis have increased rapidly in the past few years, and the commonly recognized gut microbiota play important roles in the regulation of anxiety^8^, depression^9^, cognition^10^ and pain^11,12^. Short-term intragastric treatment of adult mice with an antibiotic mixture impaired their novel object recognition memory by disrupting the bacterial community in the colon^13^. Exposure to a social disruption stressor changed the structure of the colonic mucosa-associated microbiota in mice and resulted in anxiety-like behavior^14^. In addition, comorbid gastrointestinal (GI) inflammation accompanies humoral immunity to food antigens early during the course of schizophrenia^15^, and the compromised endothelial barrier^16^ and permeability of the blood–brain barrier lead to translocation of food-derived peptides and resident gut microbiota^17^.

METH-mediated release of neurotransmitters might cause enteric neuron damage and death and subsequent GI dysfunction by leading to the generation of oxidative stress molecules, including reactive oxygen (ROS) and nitrogen (RNS) species^18–20^. METH use may also cause GI vasoconstriction and bowel ischemia, ultimately leading to abdominal or stomach cramping, severe constipation and/or diarrhea and tissue dehydration^21–23^. METH use is associated with ischemic colitis^24–26^ and vasculitis of the distal colon^27^, which may reflect inflammation caused by disruption of the intestinal barrier. METH also alters two major tight junction proteins, claudin-1 and ZO-1, which regulate gut epithelial paracellular permeability to alter colon barrier integrity^28^. Thus, the gut microbiome may also be influenced in the METH-treated animals or abusers because of these alterations in the intestine. Among men who use METH and also have sex with men, the overall composition of the gastrointestinal microbiome was altered^29^. It was reported that METH increased microbial diversity^30^, elevated the pathogenic bacteria^31^ and altered the composition of gut bacteria^32^ and metabolites in animals^33^. However, the fecal microbiota composition in heterosexual or Asian METH users remains unknown.

Changes in intestinal microbiota may influence behavior and alleviate neuropsychiatric conditions. Germ-free mice exhibited anxiolytic-like behavior in an elevated plus-maze accompanied by changes in the mRNA and protein levels of genes implicated in anxiety and stress reactivity, such as NR2B, BDNF and 5HT1A^34^. The human milk oligosaccharides 3′-sialylactose (3'SL) or 6′-sialylactose (6′SL) could attenuate stressor-induced anxiety-like behavior by supporting normal microbial communities^14^, and probiotic treatment could reverse the reduction in swim behavior and increase in immobility in maternal separation rats in a forced swim test^35^. These findings suggest that the intestinal microflora plays an important role in the regulation of cognition and mood. The therapeutic application of probiotic bacteria for the neuropsychiatric conditions caused by METH use still has broad prospects.

Thus, in this study, 16 subjects with a clinical diagnosis of METH addiction from Wuhan Mental Health Center and 14 age- and gender-matched healthy subjects were selected, and high-throughput 16S rRNA sequencing and bioinformatics analysis were performed to analyze the variations in gut microbiota between the two groups, including the differences in the diversity of the bacterial community, the microbiota composition and the taxa that best characterized each group. In addition, we analyzed the correlation between the initial age or duration of METH use and the composition of gut microbiota to determine whether the dosage of METH influenced the gut microbiota. Furthermore, the correlations between the neuropsychiatric conditions and cognitive impairments caused by METH use and the changes in gut microbiota were also a major focus of this study.

## Materials and method

### Subject selection

The study protocol was approved by the Ethics Committee of Wuhan Mental Health Center (Wuhan, Hubei, China). All participants provided written informed consent prior to enrollment after receiving a written description of the study.

We recruited 16 subjects with a clinical diagnosis of methamphetamine use disorder (average age, 36 ± 7.8; age range 27–55), defined as the METH group hereinafter, and compared them with 14 age-matched healthy subjects who had never used METH or any other addictive drug (average age, 36 ± 9.4; age range 22–54), defined as the Ctr group hereinafter. The MUD is diagnosed based on the Diagnostic and Statistical Manual of Mental Disorders 5 (DSM-5) by the consensus of two psychiatrists (Supplementary). The two groups were named METH and Ctr, respectively. All of the subjects were male. Neither their marital status nor their level of education were significantly different. All of the subjects belonged to the same nationality (Han Chinese) and lived in Wuhan city. No antibiotics, probiotics, or prebiotics were taken in the 3 months prior to sample collection. None of the subjects were on anti-inflammatory or antioxidant drugs.

Information on METH use was also collected. The average age of initial METH use was 27.5 ± 5.82 years, with a range of 22–40 years, and the average duration of METH use was 8 ± 3.40 years, with a range of 4–15 years. The average frequency of METH use was 4 ± 0.81 days per week. The subjects in the METH group had been hospitalized 1–6 times (Table 1).

**Table 1:** Characteristics of the study population.

	METH	Ctr
Subjects (n)	16	14
Proportion of males, no. (%)	16 (100%)	14 (100%)
Age (years; means ± SD) (range)	36 (7.8) (27–55)	36 (9.4) (22–54)
Employed, no. (%)	3 (18.75%)	7 (50.00%)
**Education**		
Primary school, no. (%)	5 (31.25%)	4 (28.57%)
Middle school, no. (%)	3 (18.75%)	5 (35.71%)
High school, no. (%)	1 (6.25%)	2 (14.29%)
University or higher, No. (%)	7 (43.75%)	3 (21.43%)
Married, no. (%)	8 (50.00%)	9 (64.29%)
Age of initial METH use (years; mean ± SD) (range)	27.5 (5.82) (22–40)	NA
Duration of METH use (years; mean ± SD) (range)	8 (3.40) (4–15)	NA
Frequency of METH use (days per week; mean ± SD) (range)	4 (0.81) (2–5)	NA
Number of hospitalizations (number; mean ± SD) (range)	2 (1.45) (1–6)	NA

### Assessment of cognition and symptoms of schizophrenia

One experienced psychiatrist from Wuhan Mental Health Center performed the screening examinations. Mild cognitive impairment (MCI) and dementia were assessed using the Montreal Cognitive Assessment (MoCA). The positive and negative syndrome scale (PANSS) was used to assess schizophrenia and psychosis spectrum disorders. All patients provided written informed consent.

### Fecal sample collection and DNA isolation

Fecal samples were collected from the patients who abuse METH and the matched controls in the morning and stored at − 80 °C prior to analyses. DNA was extracted using the Magnetic Soil and Stool DNA Kit (TIANGEN, Beijing, China) according to the manufacturer’s instructions. Then, the purity and concentration of DNA were determined by 1% agarose gel electrophoresis. Sample DNA was diluted to 1 ng/μL with sterile water.

### Polymerase chain reaction (PCR)

16S rRNA genes were amplified using the specific primers with barcodes (Primer: 16S V4: 515F-806R). All PCRs were carried out in 30 μL with 15 μL of Phusion High-Fidelity PCR Master Mix (New England Biolabs), 0.2 μM forward and reverse primers, and approximately 10 ng of template DNA. Thermal cycling consisted of initial denaturation at 98 °C for 1 min, followed by 30 cycles of denaturation at 98 °C for 10 s, annealing at 50 °C for 30 s, and elongation at 72 °C for 30 s and final extension at 72 °C for 5 min. A Bio-Rad T100 gradient PCR instrument was used.

The same volume of 1× loading buffer (containing SYBR green) was mixed with the PCR products, and electrophoresis was performed on a 2% agarose gel for detection. Samples with bright main bands between 400–450 bp were chosen for further experiments.

The PCR products were mixed in equidensity ratios. Then, the mixed PCR products were purified with a GeneJET Gel Extraction Kit (Thermo Scientific).

### Library preparation and sequencing

Sequencing libraries were generated using an Illumina TruSeq DNA PCR-Free Library Preparation Kit (Illumina, USA) with index codes added following the manufacturer’s recommendations. The library quality was assessed on a Qubit 2.0 fluorometer (Thermo Scientific) and an Agilent Bioanalyzer 2100 system. Finally, the library was sequenced on an Illumina NovaSeq platform, and 250 bp paired-end reads were generated.

### Data analysis

The raw data from the Illumina NovaSeq platform were spliced to obtain clean tags with quality control. Paired-end reads from the original DNA fragments were merged by FLASH and were assigned to each sample according to the unique barcodes^36^. Sequences were analyzed using the QIIME (Quantitative Insights Into Microbial Ecology) software package, and in-house Perl scripts were used to analyze alpha (within samples) and beta (among samples) diversity. First, reads were filtered by QIIME quality filters. Then, we used pick_de_novo_otus.py to pick operational taxonomic units (OTUs) by making an OTU table. Sequences with ≥ 97% similarity were assigned to the same OTU. We selected representative sequences for each OTU and used the RDP classifier to annotate taxonomic information for each representative sequence. Species richness is the number of bacterial species assigned on the basis of the OTUs detected in the samples. Alpha diversity was used to analyze the within-community microbial community diversity diversity^37^. To compute alpha diversity under the 97% consistency threshold for different samples, we rarified the OTU table and calculated three metrics: Chao1, which estimates the species abundance; the observed species, which estimates the number of unique OTUs found in each sample; and the Shannon index (Supplementary Table 1). Rarefaction curves were generated based on these three metrics. A Venn diagram was used to show the numbers of shared and unique OTUs between the two groups (R Software, http://www.r-project.org/, Version 3.0.3).

QIIME calculates both weighted and unweighted UniFrac distances (Qiime Software, http://qiime.org/, Version 1.9.1), which are phylogenetic measures of beta diversity. We used both weighted and unweighted UniFrac distances for principal coordinate analysis (PCoA), obtaining principal coordinates and visualizing them from complex, multidimensional data. We also calculated the Bray–Curtis UniFrac distances and visualize them by nonmetric multidimensional scaling (NMDS) (R Software, Version 2.15.3). To mine deeper into the microbial diversity data to explore the differences between the samples, significance tests were conducted with statistical analysis methods, including MetaStat (R Software, Version 2.15.3), LEfSe (LEfSe Software, Version 1.0) and ADONIS (R Software, Version 2.15.3). Correlations between variables were calculated using Spearman’s rank-correlation analysis (R Software, Version 2.15.3). All tests for significance were two sided, and *p* < 0.05 was considered significant.

### Ethics approval and consent to participate

The study was conducted after the approval by Wuhan Mental Health Center Ethics Committee at January 2019, and all written informed consents were obtained from the enrolled subjects, in compliance with national legislation and the Code of Ethical Principles for Medical Research Involving Human Subjects of the World Medical Association (Declaration of Helsinki). This program belongs to the Clinical cohort study on addiction-related disorders in China and registered in Chinese Clinical Trial Registry (ChiCTR2000032198).

## Results

### Diversity and community composition of gut microbiota

To characterize the gut microbiota associated with METH use disorder, the alpha diversity index was analyzed. The trend in species richness in patients with METH use disorder was similar to that in the control individuals, based on the rarefaction analysis estimates (Supplementary Fig. 1a). There were no significant differences between the METH and Ctr groups either in community richness or the number of observed species. However, the significant difference in the Shannon index showed that the Ctr group had greater species diversity (Wilcoxon rank-sum test, *p* = 0.025) (Supplementary Fig. 1b). There were 611 shared OTUs, 118 OTUs in the METH group only and 126 OTUs in the Ctr group only (Supplementary Fig. 1c).

**Figure 1:**
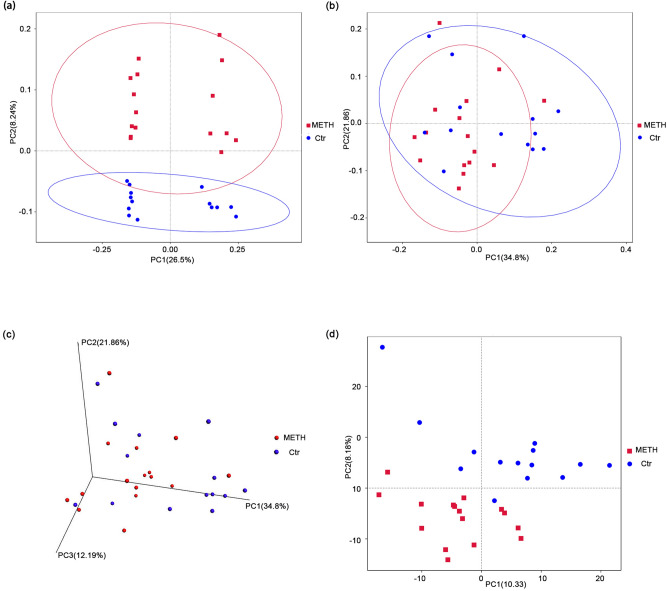
Subjects who abuse METH harbor an altered bacterial gut microbiota. PCoA of the bacterial beta diversity based on the (**a**) unweighted and (**b**) weighted UniFrac distances. (**c**) Three-dimensional PCoA of bacterial beta diversity based on the weighted UniFrac distances. (**d**) PCA of bacterial beta diversity based on the Euclidean distances. Subjects who abuse METH and Ctr subjects are colored in red and blue, respectively.

Beta diversity is used to compare the microbial community composition of different samples^38^. The beta diversity calculated on the basis of the weighted UniFrac distances, the unweighted UniFrac distances and the Bray–Curtis distances revealed a strong effect of METH use disorder on the gut microbiota^39,40^. The METH samples clustered distinctly from the controls in PCoA space and differed significantly in composition (Supplementary Table 2, with ADONIS R = 0.074, *p* = 0.013, unweighted UniFrac; ADONIS R = 0.078, *p* = 0.041, weighted UniFrac), indicating robust differences in the membership of gut bacteria between the METH and Ctr groups (Fig. 1a–c). The Bray–Curtis distances also showed significant differences between the METH and Ctr groups (Supplementary Table 2, with ADONIS R = 0.093, *p* = 0.006). Principal component analysis (PCA) based on Euclidean distances, which extracts the most important elements and structures in the data and shows the difference in multidimensional data on a two-dimensional coordinate chart^41^, also describes the similarity of the samples in the same group and the disparity between the two groups (Fig. 1d).

### Composition of gut microbiota in the METH and control groups

**Figure 2:**
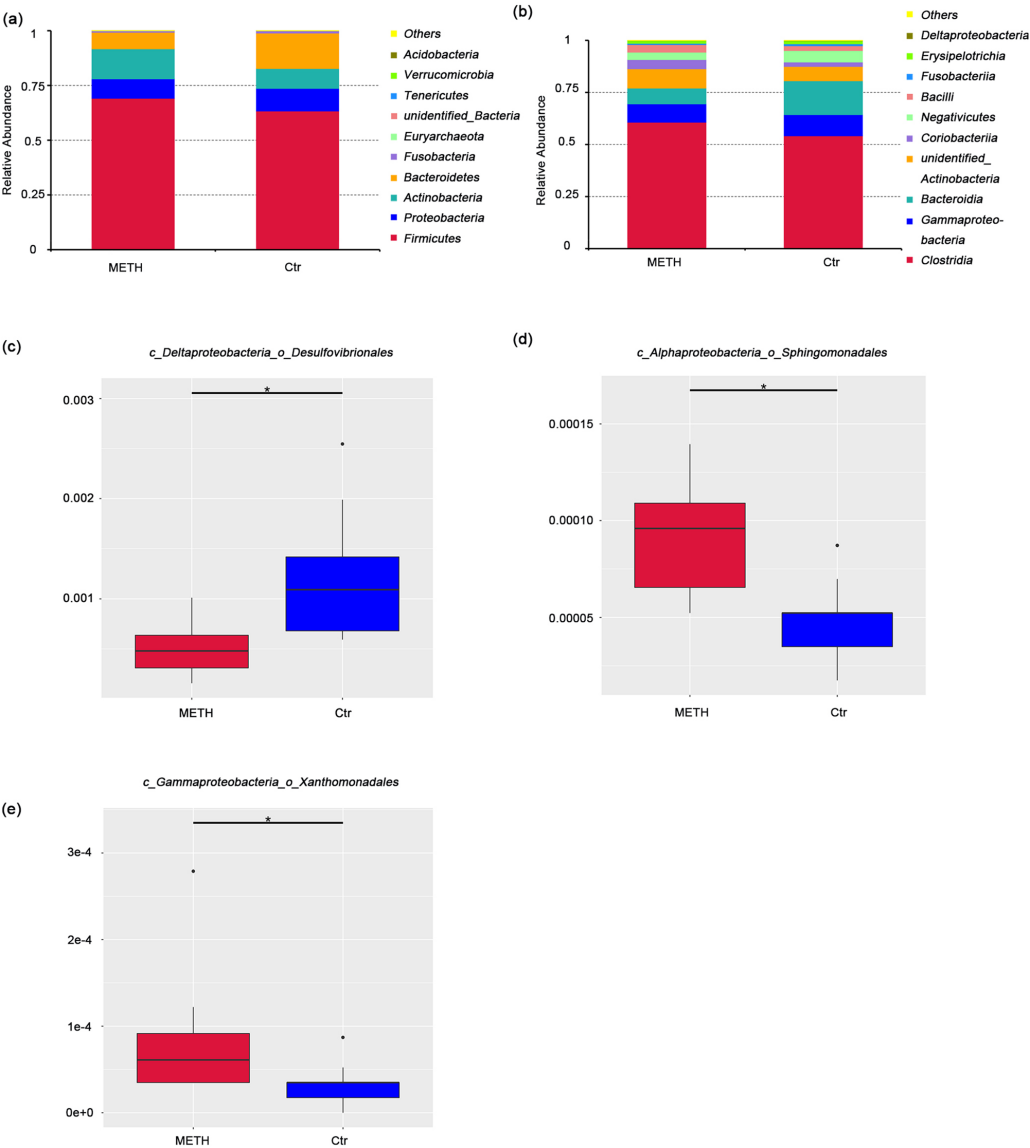
Composition of gut microbiota in the METH and control groups. Relative abundances of (**a**) phylum-level and (**b**) class-level gut microbial taxa. At the order level, (**c**) *Desulfovibrionales* was significantly decreased, and (**d**) *Sphingomonadales* and (**e**) *Xanthomonadales* were significantly increased in the METH group. *q < 0.05 (MetaStat).

The overall microbial compositions of the METH and Ctr groups were examined at different taxonomic levels. The top five most abundant phyla were *Firmicutes*, *Proteobacteria, Actinobacteria, Bacteroidetes* and *Fusobacteria* (Fig. 2a, Supplementary Table 2)*.* The top five most abundant classes were *Clostridia*, *Gammaproteobacteria*, *Bacteroidia*, unidentified *Actinobacteria* and *Coriobacteriia* (Fig. 2b, Supplementary Table 2). The results showed no phyla or classes taxa with significant differences between the two groups. At the order level, *Desulfovibrionales* was significantly decreased (MetaStat, *p* = 0.001, q = 0.021; Fig. 2c, Supplementary Table 3) and *Sphingomonadales* (MetaStat, *p* = 0.001, q = 0.021; Fig. 2d, Supplementary Table 3) and *Xanthomonadales* (MetaStat, *p* = 0.001, q = 0.021; Fig. 2e, Supplementary Table 3) were significantly increased in the METH group, but there were no significant differences in the dominant bacteria (top 10 most abundant) between the two groups. *Clostridiales, Enterobacteriales, Bacteroidales* and *Bifidobacteriales* were the top four most abundant orders (Supplementary Table 3). We found 8 statistically significant differences between the METH and Ctr groups at the family level (Supplementary Table 3), including an increase in the relative abundances of *Rikenellaceae*, an unidentified *Clostridiales* and *Desulfovibrionaceae* and a reduction in the relative abundances of *Tannerellaceae*, *Lactobacillaceae*, *Xanthomonadaceae*, *Sphingomonadaceae* and *Peptococcaceae* in the METH group. Among the top ten most abundant families, only *Clostridiales* significantly was increased in the METH group (MetaStat, *p* = 0.001, q = 0.019; Supplementary Table 3). We also found 12 significant differences in the METH group compared with the Ctr group at the genus level (Supplementary Table 3), but the abundances of all the species were low.

**Figure 3:**
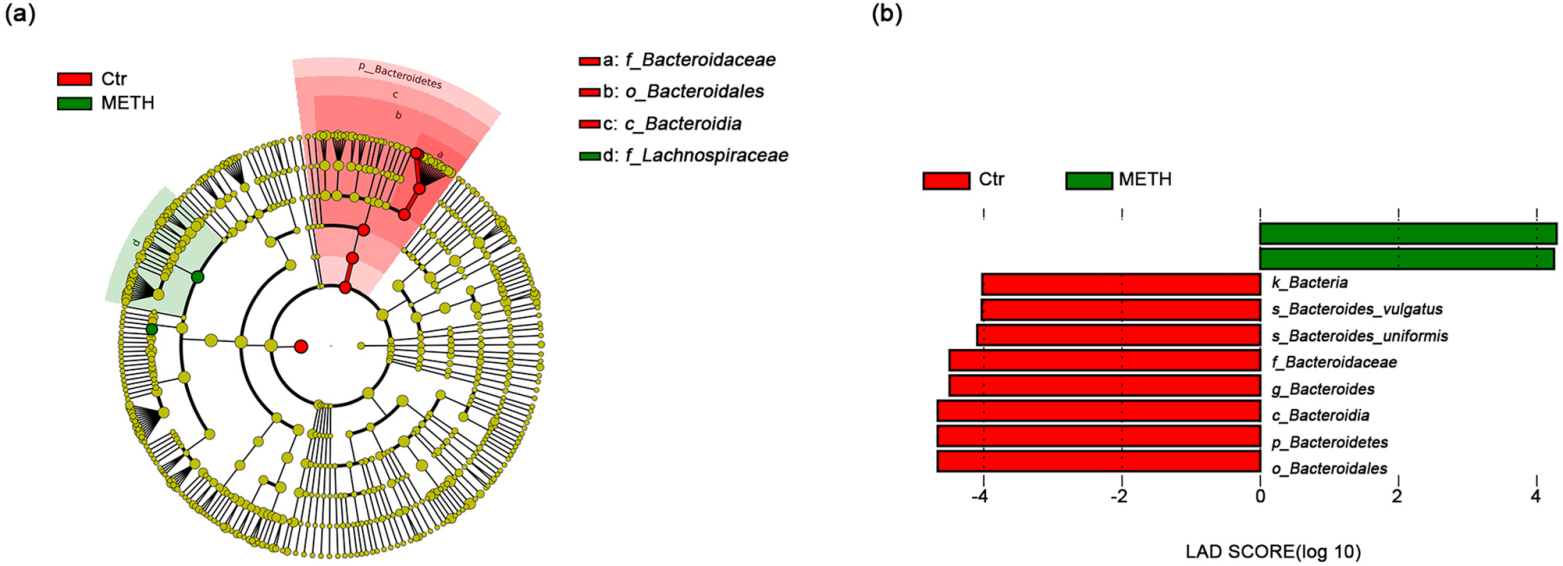
Taxonomic representation of significant differences in relative abundances between the METH and Ctr groups. (**a**) Cladograms generated by LEfSe indicating differences in the bacterial taxa between the subjects with METH use disorder (METH) and the control (Ctr) subjects. Nodes in red indicate taxa that were enriched in the Ctr compared to the METH group, while nodes in green indicate taxa that were enriched in the Ctr compared to the METH group. (**b**) LDA scores for the bacterial taxa with differential abundances between the METH and Ctr groups. Positive and negative LDA scores indicate bacterial taxa enriched in the METH and Ctr groups, respectively. Only the taxa with *p* < 0.01 (Wilcoxon rank-sum test) and LDA > 4.0 are shown in the figure legend.

We further analyzed the bacterial community structure by using the linear discriminant effect size (LEfSe), an algorithm for high-dimensional biomarker discovery that uses linear discriminant analysis (LDA) to estimate the effect size of each taxon that is differentially represented in METH and Ctr groups^33^. LEfSe analysis revealed significant increases in the relative abundances of several bacterial taxa, including *Romboutsia* and *Lachnospiraceae*, and a significant reduction in the relative abundance of *Bacteroidaceae* in the METH group compared with the Ctr group (Fig. 3a,b).

### Gut microbiota may be associated with age and the duration of METH use

**Figure 4:**
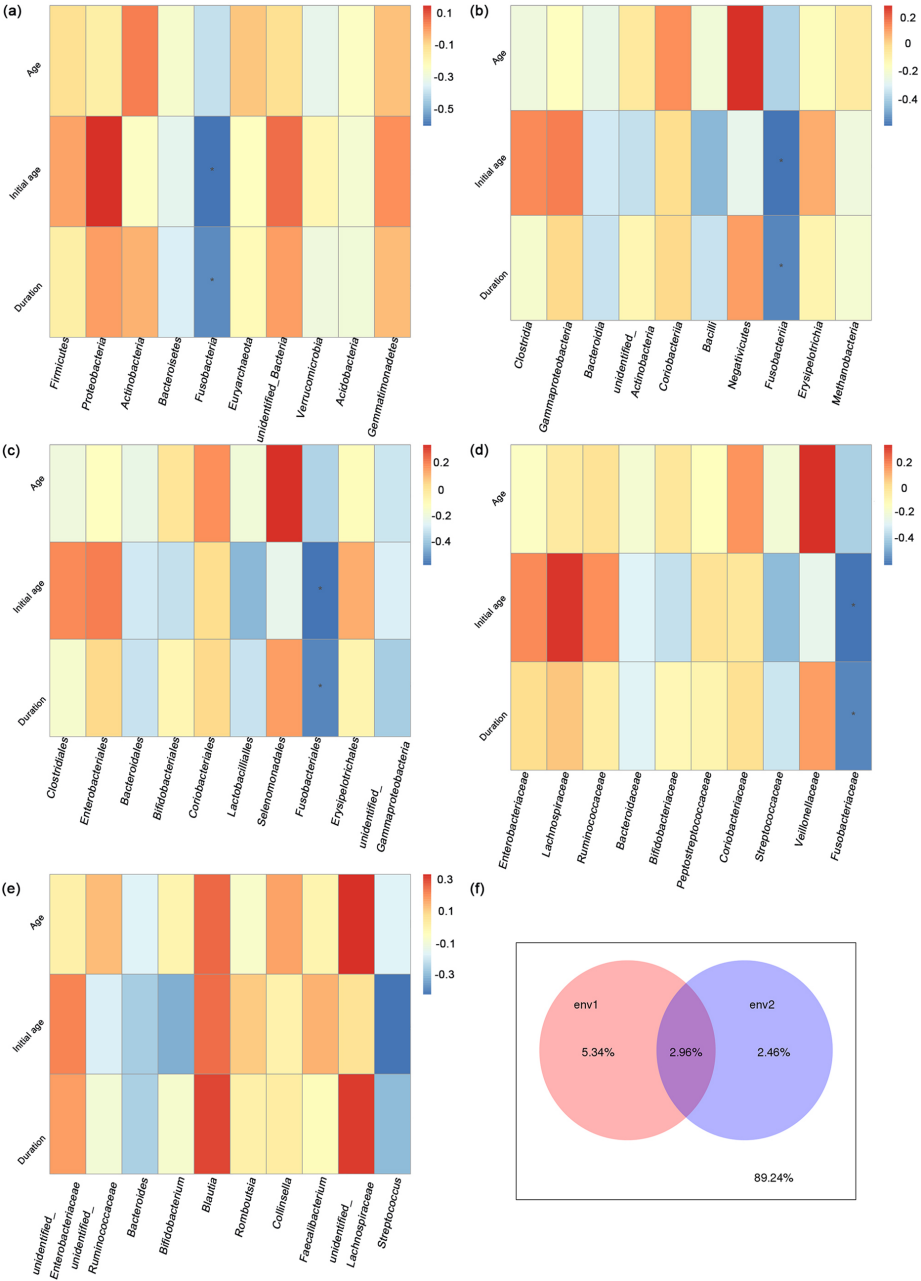
Gut microbiota may be associated with age and duration of METH use. The correlation between gut microbiota of different taxa and age, the initial age of METH use and duration of METH use (years) at the (**a**) phylum, (**b**) class, (**c**) order, (**d**) family, and e genus levels. The ordinate represents age (Age), initial age of METH use (Initial age) and duration of METH use (Duration). The abscissa represents the species information. Spearman rank correlation, *p < 0.05. (**f**) VPA showed that age (env2) contributed less to the microbial community than did the duration of METH use (env1). The intersecting part of the circle is the common explanatory quantity of env1 and env2, while the outside of the circle is the unexplainable quantity.

The above results show the influence of METH use disorder on gut microbiota but do not reveal whether the dosage of METH or the duration of METH use influences the gut microbiota. Thus, we analyzed the correlation between different taxa of the gut microbiota and age, the initial age of METH use and the duration of METH use. At the phylum level, the duration (Spearman rank correlation, R = − 0.589, *p* = 0.016) and age (Spearman rank correlation, R = − 0.545, *p* = 0.029) were both negatively related to *Fusobacteria* (Fig. 4a, Supplementary Table 4)*.* Similarly, at the class, order and family levels, the duration (Spearman rank correlation, R = − 0.589, *p* = 0.016) and age (Spearman rank correlation, R = − 0.545, *p* = 0.029) were both negatively related to *Fusobacteriia, Fusobacteriales,* and *Fusobacteriaceae*, respectively (Fig. 4b–d; Supplementary Table 4). The initial age of METH use was not significantly related to the gut microbiota. The duration was negatively related to *Streptococcus* (Spearman rank correlation, R = − 0.440, *p* = 0.088) at the genus level (Fig. 4e). Considering the interactions between microbiota and physiological aging processes^42^, we sought to determine which factor affected the microbial community distribution more, age or the duration of METH use. Variance partitioning canonical correspondence analysis (VPA) showed that the duration of METH use contributed more to the differences in the microbial community distribution than the age of the individuals did (Supplementary Fig 1, Fig. 4f.).

### Positive and negative symptoms of schizophrenia and general psychopathology and cognition were related to gut microbiota

**Figure 5:**
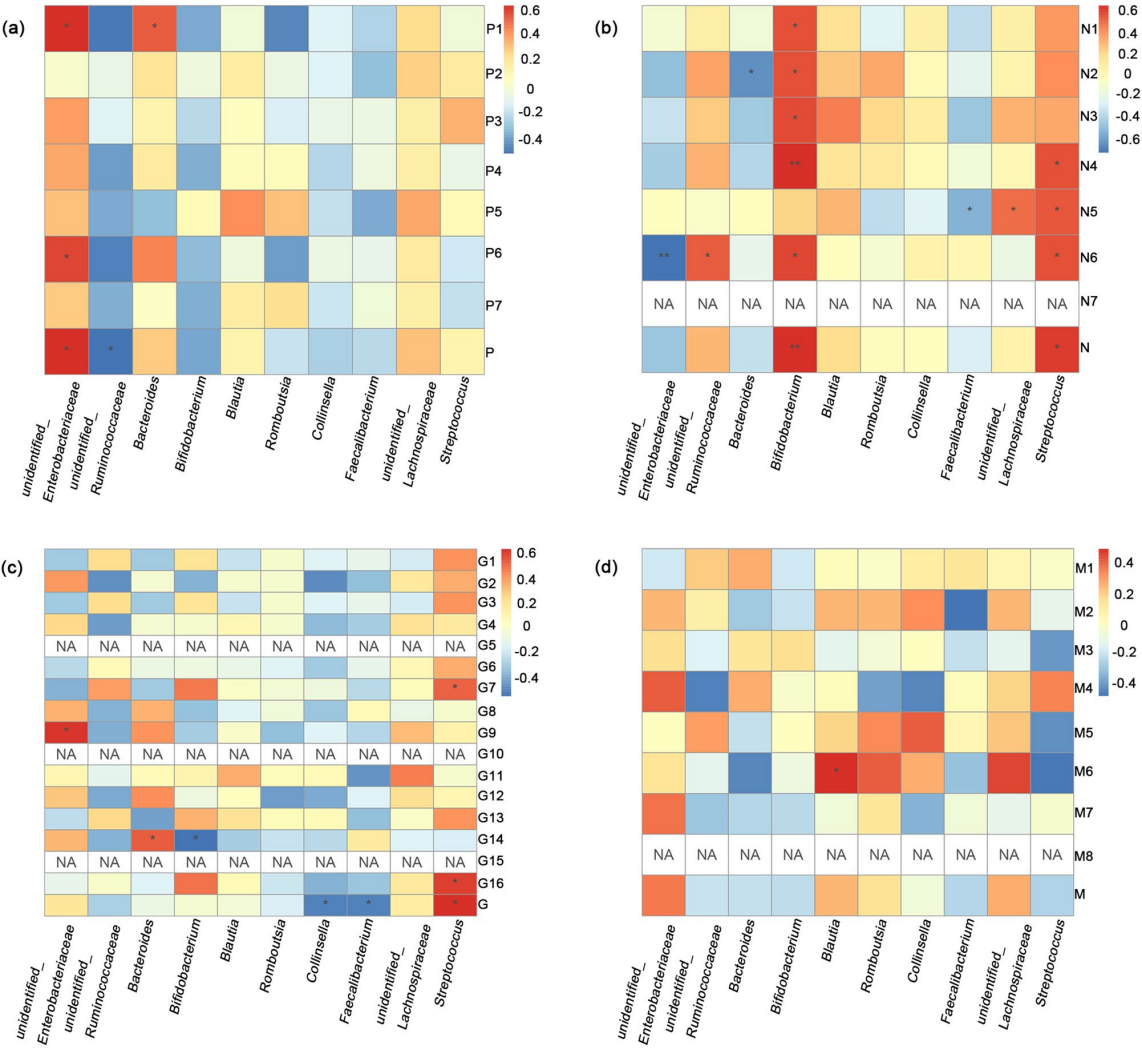
Positive and negative symptoms of schizophrenia as well as general psychopathology and cognition were related to gut microbiota. The correlations between gut microbiota at the genus level for different taxa and (**a**) the positive symptoms of schizophrenia (P1 = delusions, P2 = conceptual disorganization, P3 = hallucinatory behavior, P4 = excitement, P5 = grandiosity, P6 = suspiciousness, P7 = hostility, P = the total positive scale), (**b**) the negative symptoms of schizophrenia (N1 = blunted affect, N2 = emotional withdrawal, N3 = poor rapport, N4 = passive-apathetic social withdrawal, N5 = difficulty in abstract thinking, N6 = lack of spontaneity and flow of conversation, N7 = stereotyped thinking, N = the total negative scale) and (**c**) general psychopathology (G1 = somatic concern, G2 = anxiety, G3 = guilty feelings, G4 = tension, G5 = mannerisms and posturing, G6 = depression, G7 = motor retardation, G8 = uncooperativeness, G9 = unusual thought content, G10 = disorientation, G11 = poor attention, G12 = lack of judgment and insight, G13 = disturbance of volition, G14 = poor impulse control, G15 = preoccupation, G16 = active social avoidance, G = general psychopathology scale) from PANSS and (**d**) the MoCA (M1 = visuospatial executive, M2 = naming, M3 = attention, M4 = calculation, M5 = language, M6 = abstract, M7 = delayed recall, M8 = orientation, M = the total MoCA scale). Spearman rank correlation, *p < 0.05, **p < 0.001.

The PANSS was first developed in 1987 to assess both positive and negative symptoms of schizophrenia as well as general psychopathology^43^. METH-associated psychosis (MAP) is common and includes hallucinations and delusions^44–46^. Thus, we discuss whether phenotypes such as schizophrenia or other psychopathologies caused by METH use disorder are related to changes in gut microbiota. The positive scale showed that except for grandiosity, every index was abnormal to different degrees (Supplementary Table 5). At the genus level, an unidentified *Enterobacteriaceae* was positively related to delusions (Spearman rank correlation, R = 0.616, *p* = 0.011), suspiciousness (Spearman rank correlation, R = 0.568, *p* = 0.022) and the total positive scale (Spearman rank correlation, R = 0.620, *p* = 0.010) (Supplementary Table 6, Fig. 5a). The total positive scale was also negatively related to an unidentified *Ruminococcaceae* (Spearman rank correlation, R = − 0.507, *p* = 0.045) (Supplementary Fig 2). Delusions were positively correlated with *Bacteroides* (Spearman rank correlation, R = 0.523, *p* = 0.038) (Supplementary Table 6, Fig. 5a). Conceptual disorganization, hallucinatory behavior, excitement, grandiosity and hostility were not significantly correlated with the top ten most abundant phyla. All the individuals were absent of stereotyped thinking, and most of them received a minimal or mild level of criteria among the negative items (Supplementary Table 5). *Bifidobacterium* was positively related to blunted affect (Spearman rank correlation, R = 0.570, *p* = 0.021), emotional withdrawal (Spearman rank correlation, R = 0.561, *p* = 0.024), poor rapport (Spearman rank correlation, R = 0.582, *p* = 0.018), passive-apathetic social withdrawal (Spearman rank correlation, R = 0.652, *p* = 0.006), lack of spontaneity and flow of conversation (Spearman rank correlation, R = 0.594, *p* = 0.015) and the total negative scale (Spearman rank correlation, R = 0.650, *p* = 0.006) (Supplementary Table 6, Fig. 5b). *Streptococcus* was negatively related to passive-apathetic social withdrawal (Spearman rank correlation, R = 0.568, *p* = 0.022), difficulty in abstract thinking (Spearman rank correlation, R = 0.548, *p* = 0.028), lack of spontaneity and flow of conversation (Spearman rank correlation, R = 0.565, *p* = 0.023) and the total negative scale (Spearman rank correlation, R = 0.616, *p* = 0.011) (Supplementary Table 6, Supplementary Fig 3, Fig. 5b). There was a negative correlation between emotional withdrawal and *Bacteroides* (Spearman rank correlation, R = − 0.619, *p* = 0.011). Difficulty in abstract thinking was negatively related to *Faecalibacterium* (Spearman rank correlation, R = − 0.510, *p* = 0.044) and positively related to an unidentified *Lachnospiraceae* (Spearman rank correlation, R = 0.501, *p* = 0.048) (Supplementary Table 6, Fig. 5b). Lack of spontaneity and flow of conversation was negatively related to an unidentified *Enterobacteriaceae* (Spearman rank correlation, R = − 0.714, *p* = 0.002) and positively related to an unidentified *Ruminococcaceae* (Spearman rank correlation, R = 0.537, *p* = 0.032) (Supplementary Table 6, Supplementary Fig. 4, Fig. 5b). For the general psychopathology scale, the results for mannerisms and posturing, for disorientation and for preoccupation all showed no abnormality (Supplementary Table 5). *Fusobacteria* was positively related to motor retardation (Spearman rank correlation, R = 0.510, *p* = 0.044), active social avoidance (Spearman rank correlation, R = 0.573, *p* = 0.020) and the total general psychopathology scale (Spearman rank correlation, R = 0.617, *p* = 0.011) (Supplementary Table 6, Fig. 5c). Unusual thought content was positively related to an unidentified *Enterobacteriaceae* (Spearman rank correlation, R = 0.606, *p* = 0.013) (Supplementary Table 6, Fig. 5c). Poor impulse control was negatively related to *Bifidobacterium* (Spearman rank correlation, R = − 0.545, *p* = 0.029) and positively related to *Bacteroides* (Spearman rank correlation, R = 0.522, *p* = 0.038) (Supplementary Table 6, Fig. 5c). The total general psychopathology scale was negatively related to *Collinsella* (Spearman rank correlation, R = − 0.504, *p* = 0.047) and *Faecalibacterium* (Spearman rank correlation, R = − 0.502, *p* = 0.047) (Supplementary Table 6, Fig. 5c) (Supplementary Fig 5).

The MoCA, developed by Nasreddine et al. in 2005, is widely used to facilitate the detection of MCI^39^. We also analyzed the correlation between the scales of MoCA and the gut microbiota at the genus level. *Blautia* was positively related to abstraction (Spearman rank correlation, R = 0.502, *p* = 0.047) (Supplementary Table 6, Fig. 5d) (Supplementary Fig 5).

## Discussion

This study of 16 individuals with METH use disorder and 14 age-matched subjects found that METH use significantly impacted gut microbial composition, and the impacts were related to the age of the individual and the duration of METH use. We found differences in the Shannon index, which indicated greater bacterial diversity in the Ctr group. However, greater fecal microbial diversity (estimated using the Shannon and Simpson indices) in METH CPP rats has been reported before^30^. The strict control over the animal-related variables, including the living environment, age, sex, etc., may have contributed greatly to these results. The nonnegligible differences in the duration of drug use also affected the gut microbiota. The average duration of METH use in the study subjects was 8 years, far longer than that in the METH CCP rats. In addition, many studies have shown that in adults, higher microbiota diversity correlates with increased overall healt^42^.

In this study, changes in gut microbial community composition were identified from the beta diversity differences between the two groups assessed via ADONIS analysis of the weighted UniFrac, unweighted UniFrac and Bray–Curtis distances. A cohort study from the University of California also showed significant variation in the overall gut microbiota composition of METH users^29^. In addition, gut dysbiosis was characterized by significant taxonomical differences between one of the major classes and three of the orders in METH users versus control subjects. *Deltaproteobacteria* abundance was reduced in the METH group and has also been reported to be reduced with chronic inflammation of the gastrointestinal (GI) tract and Crohn’s disease^47^, and there were also cases of chronic METH use with presentations of the signs and symptoms of Crohn's disease^48^. Furthermore, we discovered that the relative abundances of the orders *Sphingomonadales* and *Xanthomonadales* were significantly increased in the gut microbiota of the subjects in the METH group compared to that of the control subjects, while the relative abundance of the order *Desulfovibrionales* was significantly reduced in the individuals in the METH group.

Cases of people who chronically use METH and suffer from gastrointestinal diseases, such as nonocclusive mesenteric ischemia^48^ and intestinal ischemia^49^, may help explain these changes in the gut microbiota composition. Our results showed that several gut microorganisms, including *Fusobacteria, Fusobacteriia, Fusobacteriales* and *Fusobacteriaceae*, were negatively correlated with the duration of METH use. *Fusobacteria* are anaerobic gram-negative bacilli that are natural constituents of the microbiota in the mouth and other mucosal sites in humans^50^. However, several *Fusobacteria* taxa were significantly more abundant in METH CPP rats^30^, indicating the different influence between chronic and short-term METH use on gut microbiota. *Fusobacteria* and *Fusobacteriia* were both reported to increase in the stool microbiome of individuals with colorectal cancer compared with healthy individuals^51,52^. Patients with Crohn's disease showed an increased abundance of *Fusobacteriaceae*^53^. The role of those bacteria in the pathogenesis of gastrointestinal tract disease or gastrointestinal tract impairment related to METH use is unknown. The positive correlations between *Fusobacteria* and motor retardation, active social avoidance and the total general psychopathology scale may also indicate its important role in METH use disorders. Although age was negatively related to *Fusobacteria*, *Fusobacteriia*, *Fusobacteriales*, *Fusobacteriaceae* and *Fusobacterium* at different taxonomic levels in both the METH and Ctr groups, there are no studies about the relationship between those bacteria and aging (Supplementary Table 5). Therefore, these are potential new correlations of microbiota with aging among Asians in both METH users and non-drug users. VPA showed that the duration of METH use contributed more to the differences in microbial community distribution than did the age of the individuals, so the role of METH could not be ignored.

METH can induce psychosis and even a persistent psychotic syndrome that shows similarities to schizophrenia among recreational and chronic users^54^. Chronic METH users have higher rates of deficits in memory, executive functioning, anxiety, depression, and most notably psychosis^48^. The METH users in our study also showed abnormalities in the PANSS and MoCA tests (Supplementary Table 5). A higher abundance of the *Enterobacteriaceae* family triggers colonic inflammatory conditions and is distinctly increased in the colon of patients with non-tremor-dominant Parkinson disease (PD)^55,56^. *P. mirabilis*, belonging to the *Enterobacteriaceae* family, worsened motor symptoms and dopaminergic neuronal damage in mice at the premotor phase of PD^57^, which may be related to the positive symptoms of schizophrenia induced by METH use in the same way (Supplementary Table 6). The abundance of *Ruminococcaceae* was reported to be correlated with a lower severity of negative symptoms^58^ and was found to be negatively related to the total positive scale for the METH users in our study (Supplementary Table 6). Although the relative abundances of fecal *Bacteroides* were reported to be negatively correlated with brain signatures associated with depression^59^, we found that *Bacteroides* was positively correlated with delusions and poor impulse control but negatively correlated with emotional withdrawal (Supplementary Table 6). GABA could be produced by some *Bacteroides*^59^, and its uptake and release were reduced in cortical synaptosomes prepared from schizophrenic brains^60^, which may be related to the positive correlation we found in the present study (Supplementary Table 6). *Bifidobacterium longum* 1714™ has been shown to reduce stress-related behaviors in preclinical studies and improve stress responses^61,62^ and cognitive function in healthy volunteers^63^, but *Bifidobacterium* was positively related to several items of the negative scale and poor impulse control in our study (Supplementary Table 6). *Faecalibacterium* was reported to be negatively correlated with the severity of depressive symptoms^64^ and negatively related to difficulty in abstract thinking in this study, which may be influenced by its butyrate-producing function^65^. Although opposite trends of *Enterobacteriaceae* and *Faecalibacterium* abundance were found in patients with major depressive disorder^59^, these bacteria were all negatively correlated with the items of the negative scale in METH users (Supplementary Table 6). *Enterobacteriaceae* and *Faecalibacterium* were also correlated with the items of the general psychopathology scale in METH users (Supplementary Table 6). Furthermore, *Lachnospiraceae* and *Ruminococcaceae,* which were positively related to the items of the negative scale (Supplementary Table 6), were all found to be decreased in the responding individuals with major depressive disorder^59^. *Blautia,* which was positively related to abstraction in the cognition assessment (Supplementary Table 6), was also found to be increased in patients with major depressive disorder^64,66^.

Our results showed alterations in gut microbiota composition and significantly different taxa at various levels in METH users and age-matched individuals. However, the absence of diet data and the unique ethnicity, gender and sexual orientation may limit the generalizability of our findings to other groups. Fortunately, this work could supplement the research reporting the gut microbiota of male METH users who had sex with men^29^. Although not all of the altered genera were the same in their research and ours, the reduction in *Bacteroides* was similar and significant^29^. Considering the differences in ethnicity and sexual behavior patterns, the variation in the results was acceptable.

Here, we observed an altered intestinal microbial community, including richer bacterial diversity and significant changes in the relative abundance of several genera, in individuals who abused METH. These features may be associated with impairment of the gastrointestinal tract in chronic METH users. In addition, the genera that were significantly related to the duration of METH use highlight the chronic effect of this drug. There is currently no accepted pharmaceutical treatment for psychosis induced by METH. Thus, further research on gut microbiota alterations in people who abuse METH, especially those with symptoms of schizophrenia or cognitive impairment, may inform therapeutic approaches. The common altered microbes in psychosis induced by METH and other factors also revealed potential relationships with pathogenesis.

## Supplementary Information

Supplementary Information.

Supplementary Figure 1.

Supplementary Figure 2.

Supplementary Figure 3.

Supplementary Figure 4.

Supplementary Figure 5.

## Data Availability

All datasets generated for this study are included in the manuscript/supplementary files. The data have already been uploaded to SRA(ID: PRJNA679237).
