# Supplementary material for: Altered fecal microbiota composition in individuals who abuse methamphetamine
Source: Sci Rep. 2021 Sep 13;11:18178. doi: 10.1038/s41598-021-97548-1 (PMC8437956; doi:10.1038/s41598-021-97548-1)
Supplement: Supplementary file 1 — Supplementary Information. [file 41598_2021_97548_MOESM1_ESM.pdf]

Supplementary Fig 1 Overall structure of the fecal bacterial communities.

(a) The rarefaction curve of the number of OTUs. The x-coordinate is the number of sequencing bars randomly selected from a sample, and the y-coordinate is the number of OTUs that can be constructed based on the sequencing bars, which is used to reflect the depth of sequencing. Different samples are represented by different-colored curves. **(b)** Rank abundance of the METH and Ctr groups. The x-coordinate is the ordinal number sorted by OTU abundance, and the y-coordinate is the relative abundance of the corresponding OTUs. Different samples are represented by dashed lines of different colors. The FB samples belong to the METH group; the FBC samples belong to the Ctr group. **(c)** Venn diagram based on OTU clustering. Each circle represents a group, the number of OTUs in the overlapping regions of the circles represents the number of common OTUs between the two groups, and the number of OTUs in non-overlapping regions represents the number of unique OTUs in each group.

Supplementary Fig 2 Gut microbiota may be associated with age and the duration of METH use. (a), (c), (e), (g) Correlation between age and the relative abundance of the taxa in phylum, class, order and family level respectively. **(b), (d), (f), (h)** Correlation between duration of the METH use and the relative abundance of the taxa in phylum, class, order and family level respectively.

Supplementary Fig 3 Positive symptoms of schizophrenia were related to gut microbiota. (a)-(e) Bacterial taxa were correlated with delusions, suspiciousness and total positive scale respectively.

Supplementary Fig 4 Negative symptoms of schizophrenia were related to gut microbiota. (a)-(o) Bacterial taxa were correlated with blunted affect, emotional withdrawal, passive-apathetic social withdrawal, difficulty in abstract thinking, lack of spontaneity & flow of conversation and total negative scale respectively.

Supplementary Fig 5 General psychopathology and cognition were related to gut microbiota were related to gut microbiota. (a)-(h) Bacterial taxa were correlated with motor retardation, unusual thought content, poor impulse control, active social avoidance, general psychopathology scale and abstraction respectively.

We mainly recruited the patients with methamphetamine use disorder (MUD) in our research. The MUD is diagnosed based on the Diagnostic and Statistical Manual of Mental Disorders 5 (DSM-5) by the consensus of two psychiatrists. The details are as followed:

The Diagnostic Criteria of MUD

A pattern of amphetamine-type substance, cocaine, or other stimulant use leading to clinically significant impairment or distress, as manifested by at least two of the following, occurring within a 12-month period:

1. The stimulant is often taken in larger amounts or over a longer period than was intended.
2. There is a persistent desire or unsuccessful efforts to cut down or control stimulant use.
3. A great deal of time is spent in activities necessary to obtain the stimulant, use the stimulant, or recover from its effects.
4. Craving, or a strong desire or urge to use the stimulant.
5. Recurrent stimulant use resulting in a failure to fulfill major role obligations at work, school, or home.
6. Continued stimulant use despite having persistent or recurrent social or inteφersonal problems caused or exacerbated by the effects of the stimulant.
7. Important social, occupational, or recreational activities are given up or reduced because of stimulant use.
8. Recurrent stimulant use in situations in which it is physically hazardous.
9. Stimulant use is continued despite knowledge of having a persistent or recurrent

physical or psychological problem that is likely to have been caused or exacerbated by the stimulant.

10. Tolerance, as defined by either of the following:

a. A need for markedly increased amounts of the stimulant to achieve intoxication or desired effect.

b. A markedly diminished effect with continued use of the same amount of the stimulant.

Note: This criterion is not considered to be met for those taking stimulant medications solely under appropriate medical supervision, such as medications for attention-deficit/hyperactivity disorder or narcolepsy.

11. Withdrawal, as manifested by either of the following:

a. The characteristic withdrawal syndrome for the stimulant (refer to Criteria A and B of the criteria set for stimulant withdrawal, p. 569).

b. The stimulant (or a closely related substance) is taken to relieve or avoid withdrawal symptoms.

Supplementary Table 1. Comparison of alpha diversity under the 97% consistency threshold in the two goups.

group	observed_species	shannon	simpson	chao1	ACE	goods_coverage
Ctr	325	5.206	0.932	369.952	382.298	0.999
METH	306	4.935*	0.922	342.294	354.654	0.999

cutoff=57339

Wilcoxon rank-sum test, *p<0.05

Supplementary Table 2. Relative abundance of top ten microbes of different levels in two groups.

phylum

Taxonomy	METH	Ctr
Firmicutes	0.691129	0.633175
Proteobacteria	0.089046	0.103454
Actinobacteria	0.13697	0.09035
Bacteroidetes	0.075762	0.162449
Fusobacteria	0.006247	0.009618
Euryarchaeota	0.00017	0
unidentified_Bacteria	0.000343	0.000443
Tenericutes	0.000013	0.000072
Verrucomicrobia	0.000081	0.000035
Acidobacteria	0.000087	0.000096
Others	0.000152	0.000308

class

Taxonomy	METH	CTR
Clostridia	0.606823	0.541457
Gammaproteobacteria	0.088193	0.102084
Bacteroidia	0.075668	0.162105
unidentified_ Actinobacteria	0.092859	0.069014
Coriobacteriia	0.043815	0.021174
Negativicutes	0.035908	0.055461
Bacilli	0.036147	0.022614
Fusobacteriia	0.006238	0.009607
Erysipelotrichia	0.012575	0.014011
Deltaproteobacteria	0.000543	0.001186
Others	0.001232	0.001285

order

Taxonomy	METH	CTR
Clostridiales	0.606812	0.541431
Enterobacteriales	0.08599	0.094846
Bacteroidales	0.075648	0.162093
Bifidobacteriales	0.092374	0.068532
Coriobacteriales	0.043811	0.021173
Selenomonadales	0.035908	0.055461
Lactobacillales	0.036059	0.022525
Fusobacteriales	0.006238	0.009607
Erysipelotrichales	0.012575	0.014011
unidentified_ Gammaproteobacteria	0.001267	0.00683
Others	0.003319	0.003491

family

Taxonomy	METH	CTR
Lachnospiraceae	0.321415	0.286737
Enterobacteriaceae	0.08599	0.094846
Ruminococcaceae	0.181135	0.188753
Bacteroidaceae	0.059185	0.116566
Bifidobacteriaceae	0.092374	0.068532
Peptostreptococcaceae	0.079787	0.054748
Coriobacteriaceae	0.041497	0.019457
Veillonellaceae	0.03235	0.054599
Streptococcaceae	0.032094	0.021339
Fusobacteriaceae	0.006238	0.009607
Others	0.067936	0.084816

genus

Taxonomy	METH	CTR
unidentified_Enterobacteriaceae	0.071641	0.083155
unidentified_ Ruminococcaceae	0.056424	0.041479
Bacteroides	0.059185	0.116566
Blautia	0.117765	0.10988
Bifidobacterium	0.092346	0.068528
Faecalibacterium	0.079266	0.106997
Romboutsia	0.073408	0.039146
Collinsella	0.041485	0.019456
Megamonas	0.006586	0.012668
unidentified _Lachnospiraceae	0.071749	0.059381
Others	0.330146	0.342746

species

Taxonomy	METH	CTR
Escherichia_ coli	0.071641	0.083155
Ruminococcus_bromii	0.043127	0.019636
Bifidobacterium_pseudocatenulatum	0.082387	0.051101
Collinsella_ aerofaciens	0.041114	0.018949
Ruminococcus_sp_5_1_39BFAA	0.078941	0.064517
Bacteroides_ plebeius	0.016	0.002601
Dialister _succinatiphilus	0.004137	0.01129
Fusobacterium_sp_RMA_1065	0.005827	0.009355
[Ruminococcus]_gnavus	0.014659	0.010364
Bacteroides_ vulgatus	0.015938	0.035737
Others	0.626229	0.693297

Supplementary Table 3. MetaStat of microbes with significant difference between the METH and Ctr groups.

class

Taxa	mean (Ctr)	variance (Ctr)	standard error(Ctr)	mean (METH)	variance (METH)	standard error (METH)	p value	q value
k__Bacteria;p__Proteobacteria; c__Deltaproteobacteria;	0.001168489	3.18E-07	0.00015079	0.000530834	7.37E-08	6.79E-05	0.000999	0.028971

Order

Taxa	mean(Ctr)	variance(Ctr)	standard error(Ctr)	mean(METH)	variance (METH)	standard error (METH)	p value	q value
k__Bacteria;p__Proteobacteria;c__Deltaproteobacteria;o__Desulfovibrionales;	0.001159769	3.19E-07	0.00015102	5.09E-04	6.63E-08	6.44E-05	0.000999	0.020979
k__Bacteria;p__Proteobacteria;c__Gammaproteobacteria;o__Xanthomonadales;	3.11E-05	5.23E-10	6.11E-06	7.74E-05	3.85E-09	1.55E-05	0.000999	0.020979
k__Bacteria;p__Proteobacteria;c__Alphaproteobacteria;o__Sphingomonadales;	5.11E-05	3.96E-10	5.32E-06	9.16E-05	9.53E-10	7.72E-06	0.000999	0.020979

Family

Taxa	mean(Ctr)	variance(Ctr)	standard error(Ctr)	mean(METH)	variance (METH)	standard error (METH)	p value	q value
k__Bacteria;p__Bacteroidetes;c__Bacteroidia;o__Bacteroidales;f__Rikenellaceae;	0.020880827	0.000488668	0.00590803	3.33E-03	6.24E-06	6.25E-04	0.000999	0.018815
k__Bacteria;p__Firmicutes;c__Clostridia;o__Clostridiales;f__unidentified_ Clostridiales;	6.72E-03	1.88E-05	1.16E-03	2.19E-02	2.44E-04	3.90E-03	0.000999	0.018815
k__Bacteria;p__Bacteroidetes;c__Bacteroidia;o__Bacteroidales; f__Tannerellaceae;	1.63E-02	1.53E-04	3.31E-03	4.80E-03	1.74E-05	1.04E-03	0.001998	0.032253
k__Bacteria;p__Firmicutes;c__Bacilli;o__Lactobacillales;f__Lactobacillaceae;	8.99E-04	1.87E-07	1.15E-04	3.73E-03	6.36E-06	6.30E-04	0.000999	0.018815
k__Bacteria;p__Proteobacteria;c__Deltaproteobacteria;o__Desulfovibrionales; f__Desulfovibrionaceae;	1.16E-03	3.19E-07	1.51E-04	5.09E-04	6.63E-08	6.44E-05	0.000999	0.018815
k__Bacteria;p__Proteobacteria;c__Gammaproteobacteria;o__Xanthomonadales; f__Xanthomonadaceae;	3.11E-05	5.23E-10	6.11E-06	7.30E-05	2.52E-09	1.26E-05	0.002997	0.042333
k__Bacteria;p__Proteobacteria;c__Alphaproteobacteria; o__Sphingomonadales; f__Sphingomonadaceae;	5.11E-05	3.96E-10	5.32E-06	9.16E-05	9.53E-10	7.72E-06	0.000999	0.018815
k__Bacteria;p__Firmicutes;c__Clostridia; o__Clostridiales;f__Peptococcaceae;	0.00E+00	0.00E+00	0.00E+00	1.31E-05	5.47E-10	5.85E-06	0.000636	0.018815

Genus

Taxa	mean(Ctr)	variance(Ctr)	standard error(Ctr)	mean(METH)	variance (METH)	standard error (METH)	p value	q value
k__Bacteria;p__Bacteroidetes;c__Bacteroidia; o__Bacteroidales;f__Rikenellaceae; g__Alistipes;	0.02078366	0.000486976	0.00589779	3.32E-03	6.23E-06	6.24E-04	0.000999	0.023876
k__Bacteria;p__Firmicutes;c__Clostridia; o__Clostridiales;f__unidentified_ Clostridiales; g__unidentified_Clostridiales;	0.006480257	1.85E-05	0.00115012	2.17E-02	2.41E-04	3.88E-03	0.000999	0.023876
k__Bacteria;p__Firmicutes;c__Clostridia;o__Clostridiales;f__Lachnospiraceae; g__Roseburia;	6.11E-03	1.64E-05	1.08E-03	0.01536912	0.0001274	0.002822	0.001998	0.034109
k__Bacteria;p__Bacteroidetes;c__Bacteroidia;o__Bacteroidales; f__Tannerellaceae;g__Parabacteroides;	1.63E-02	1.53E-04	3.31E-03	0.004798218	1.74E-05	0.001043	0.002997	0.044768
k__Bacteria;p__Proteobacteria;c__Gammaproteobacteria; o__unidentified_Gammaproteobacteria;f__Burkholderiaceae;g__Sutterella;	2.14E-03	9.62E-06	8.29E-04	0.000252882	7.92E-08	7.04E-05	0.000999	0.023876
k__Bacteria;p__Firmicutes;c__Clostridia;o__Clostridiales;f__Lachnospiraceae; g__Lachnospira;	4.07E-03	5.11E-06	6.04E-04	0.001979456	1.84E-06	0.000339	0.001998	0.034109

k__Bacteria;p__Firmicutes;c__Bacilli;o__Lactobacillales;f__Lactobacillaceae; g__Lactobacillus;	8.99E-04	1.87E-07	1.15E-04	0.003733279	6.36E-06	0.00063	0.000999	0.023876
k__Bacteria;p__Firmicutes;c__Negativicutes; o__Selenomonadales; f__Veillonellaceae;g__Mitsuokella;	6.60E-05	4.72E-08	5.80E-05	0.003471677	1.31E-05	0.000905	0.001998	0.034109
k__Bacteria;p__Bacteroidetes;c__Bacteroidia;o__Bacteroidales;f__Marinifilaceae;g__Butyricimonas;	1.08E-03	4.22E-06	5.49E-04	9.05E-05	1.07E-08	2.59E-05	0.000999	0.023876
k__Bacteria;p__Firmicutes;c__Bacilli; o__Lactobacillales;f__Streptococcaceae; g__Lactococcus;	0.000626599	5.14E-07	0.00019167	8.94E-05	1.65E-08	3.22E-05	0.000999	0.023876
k__Bacteria;p__Proteobacteria; c__Deltaproteobacteria;o__Desulfovibrionales; f__Desulfovibrionaceae;g__Desulfovibrio;	0.00058549	1.78E-07	0.00011287	0.000138431	1.17E-08	2.71E-05	0.000999	0.023876
k__Bacteria;p__Firmicutes;c__Clostridia; o__Clostridiales;f__Ruminococcaceae; g__Oscillospira;	0.000342574	1.39E-07	9.97E-05	6.98E-05	1.57E-08	3.14E-05	0.002997	0.044768
k__Bacteria;p__Firmicutes;c__Negativicutes; o__Selenomonadales; f__Veillonellaceae;g__Allisonella;	0.00052445	2.01E-07	0.00011993	7.96E-05	6.65E-09	2.04E-05	0.000999	0.023876

k__Bacteria;p__Firmicutes;c__Clostridia; o__Clostridiales;f__Ruminococcaceae; g__Negativibacillus;	0.000450952	8.13E-08	7.62E-05	0.000136251	3.44E-08	4.63E-05	0.000999	0.023876
k__Bacteria;p__Firmicutes;c__Clostridia; o__Clostridiales;f__Ruminococcaceae; g__Fournierella;	6.60E-05	6.23E-09	2.11E-05	1.20E-05	5.97E-10	6.11E-06	0.001998	0.034109
k__Bacteria;p__Proteobacteria; c__Alphaproteobacteria;o__Sphingomonadales; f__Sphingomonadaceae;g__Sphingomonas;	4.98E-05	4.61E-10	5.74E-06	8.72E-05	1.18E-09	8.57E-06	0.000999	0.023876

Supplementary Table 4. Spearman rank correlation between the microbes and the initial age of METH use, duration of METH use and age.

phylum

p value	Firmicu-tes	Proteobacteria	Actinobacteria	Bacteroidetes	Fusobacteria	Euryarchaeota	unidentified_Bacteria	Verrucomicrobia	Acidobacteria	Gemmatimonadetes
initial age of METH use	0.7558446	0.5954388	0.80198566	0.3736559	0.1487856	0.885448	0.7409473	0.2544258	0.4173	0.89467126
duration of METH use	0.9691767	0.5078123	0.42775926	0.2412082	0.0163304	0.4309328	0.7375117	0.5266131	0.34957	0.86454112
age	0.6072544	0.9392712	0.97395512	0.2031657	0.0289539	0.49822	0.9283455	0.3129951	0.32894	0.92973199

R	Firmicutes	Proteobacteria	Actinobacteria	Bacteroidetes	Fusobacteria	Euryarchaeota	unidentified_Bacteria	Verrucomicrobia	Acidobacteria	Gemmatimonadetes
initial age of METH use	-0.084447	-0.143708	0.06815002	-0.238525	-0.378066	-0.039181	-0.089764	-0.302723	-0.218	-0.0360095
duration of METH use	0.0105128	0.1787182	-0.2132603	-0.31088	-0.589152	-0.211832	0.0909948	-0.170994	-0.2504	0.04638946
age	-0.139159	0.0207258	-0.0088825	-0.336055	-0.545195	-0.18271	0.0244629	-0.269388	-0.261	-0.0239884

class

p value	Clostridia	Gammaproteobacteria	Bacteroidia	unidentified_Actinobacteria	Coriobacteriia	Bacilli	Negativicutes	Fusobacteriia	Erysipelotrichia	Methanobacteria
initial age of METH use	0.4308763	0.5954388	0.37365591	0.9132576	0.5726212	0.4508989	0.2361371	0.1487856	0.65005	0.88544797
duration of METH use	0.5334778	0.5078123	0.2412082	0.198402	0.9735785	0.0798815	0.3547822	0.0163304	0.70666	0.43093276
age	0.5032534	0.9392712	0.20316574	0.7106716	0.9306139	0.2052882	0.634514	0.0289539	0.75187	0.49821995
R	Clostridia	Gammaproteobacteria	Bacteroidia	unidentified_Actinobacteria	Coriobacteriia	Bacilli	Negativicutes	Fusobacteriia	Erysipelotrichia	Methanobacteria
initial age of METH use	-0.211858	-0.143708	-0.2385251	-0.02963	0.1525968	-0.202969	0.3140827	-0.378066	-0.123	-0.0391814
duration of METH use	0.1682053	0.1787182	-0.3108795	-0.339414	0.009011	-0.45055	-0.247803	-0.589152	0.10212	-0.2118323
age	-0.180611	0.0207258	-0.3360548	-0.100668	0.0236867	-0.334574	0.1287963	-0.545195	-0.0859	-0.1827102

order

p value	Clostridiales	Enterobacteriales	Bacteroidales	Bifidobacteriales	Coriobacteriales	Lactobacillales	Selenomonadales	Fusobacteriales	Erysipelotrichales	unidentified_Gammaproteobacteria
initial age of METH use	0.4308763	0.5954388	0.37365591	0.9565717	0.5726212	0.4508989	0.2361371	0.1487856	0.65005	0.22657193
duration of METH use	0.5334778	0.5078123	0.2412082	0.198402	0.9735785	0.0798815	0.3547822	0.0163304	0.70666	0.26806614
age	0.5032534	0.9392712	0.20316574	0.7229568	0.9306139	0.2052882	0.634514	0.0289539	0.75187	0.12938019
R	Clostridiales	Enterobacteriales	Bacteroidales	Bifidobacteriales	Coriobacteriales	Lactobacillales	Selenomonadales	Fusobacteriales	Erysipelotrichales	unidentified_Gammaproteobacteria
initial age of METH use	-0.211858	-0.143708	-0.2385251	-0.014815	0.1525968	-0.202969	0.3140827	-0.378066	-0.123	-0.3202443
duration of METH use	0.1682053	0.1787182	-0.3108795	-0.339414	0.009011	-0.45055	-0.247803	-0.589152	0.10212	-0.294576
age	-0.180611	0.0207258	-0.3360548	-0.096227	0.0236867	-0.334574	0.1287963	-0.545195	-0.0859	-0.3955625

family

p value	Enterobacteriaceae	Lachnospiraceae	Ruminococcaceae	Bacteroidaceae	Bifidobacteriaceae	Peptostreptococcaceae	Coriobacteriaceae	Streptococcaceae	Veillonellaceae	Fusobacteriaceae
initial age of METH use	0.5954388	0.9046185	0.98262227	0.4993759	0.9565717	0.654018	0.5501807	0.4887861	0.20493	0.14878556
duration of METH use	0.5078123	0.2317531	0.52977604	0.2974575	0.198402	0.9911916	0.9647758	0.0880687	0.37311	0.01633044
age	0.9392712	0.7852844	0.89175927	0.3101511	0.7229568	0.7894878	0.9652778	0.2388431	0.58047	0.02895391
R	Enterobacteriaceae	Lachnospiraceae	Ruminococcaceae	Bacteroidaceae	Bifidobacteriaceae	Peptostreptococcaceae	Coriobacteriaceae	Streptococcaceae	Veillonellaceae	Fusobacteriaceae
initial age of METH use	-0.143708	-0.032593	0.00592609	-0.182227	-0.014815	-0.121485	0.1614859	-0.186672	0.33482	-0.3780662
duration of METH use	0.1787182	0.3168868	0.16970717	-0.277839	-0.339414	0.0030037	-0.012015	-0.440037	-0.2388	-0.589152
age	0.0207258	0.0740209	-0.0370104	-0.270916	-0.096227	-0.07254	0.0118433	-0.312368	0.14952	-0.5451948

genus

p value	unidentified_Enterobacteriaceae	unidentified_Ruminococcaceae	Bacteroides	Bifidobacterium	Blautia	Romboutsia	Collinsella	Faecalibacterium	unidentified_Lachnospiraceae	Streptococcus
initial age of METH use	0.9956553	0.654018	0.49937588	0.9565717	0.3645463	0.7516856	0.5501807	0.9478961	0.22467	0.48878611
duration of METH use	0.4479855	0.4686832	0.29745751	0.198402	0.3793278	0.7316072	0.9647758	0.6216543	0.85971	0.08806874
age	0.5320905	0.6903453	0.31015105	0.7229568	0.2807905	1	0.9652778	0.857412	0.25806	0.23884307
R	unidentified_Enterobacteriaceae	unidentified_Ruminococcaceae	Bacteroides	Bifidobacterium	Blautia	Romboutsia	Collinsella	Faecalibacterium	unidentified_Lachnospiraceae	Streptococcus
initial age of METH use	0.0014815	0.1214848	-0.1822272	-0.014815	0.2429696	-0.085928	0.1614859	-0.017778	0.32149	-0.1866718
duration of METH use	0.2042493	-0.195238	-0.2778392	-0.339414	0.2357878	0.0931137	-0.012015	0.1336632	0.04806	-0.4400372
age	0.1687676	-0.10807	-0.2709164	-0.096227	0.287201	0	0.0118433	-0.048854	0.30052	-0.3123681

correlation coefficient -1<R<1, R >0 positve correlation, R<0 negative correlation.

Supplementary Table 5. Results of PANSS and MoCA tests.

Samples	Delusions	Conceptual disorganization	Hallucinatory behavior	Excitement	Grandiosity	Suspiciousness	Hostility	Positive Scale
FB1	3	3	3	7	3	4	7	30
FB2	2	1	3	3	1	4	3	17
FB3	4	6	3	3	1	4	3	24
FB4	2	3	2	1	1	4	2	15
FB5	3	4	3	6	1	4	6	27
FB6	2	3	2	6	1	3	6	23
FB7	2	3	2	5	1	3	5	21
FB8	4	3	3	6	1	5	6	28
FB13	6	5	3	7	1	5	7	34
FB14	5	4	4	6	1	5	6	31
FB15	4	3	3	3	1	5	5	24
FB16	6	3	6	6	1	5	5	32
FB17	6	3	3	6	1	6	5	30
FB19	5	3	3	4	1	6	4	26
FB20	5	2	6	5	1	6	5	30
FB12	5	3	3	5	1	5	5	27

Samples	Blunted affect	Emotional withdrawal	Poor rapport	Passive-apathetic social withdrawal	Difficulty in abstract thinking	Lack of spontaneity & flow of conversation	Stereotyped thinking	Negative Scale
FB1	2	5	4	5	4	3	1	24
FB2	2	4	3	3	4	4	1	21
FB3	5	4	5	5	5	6	1	31
FB4	3	5	3	5	4	5	1	26
FB5	1	3	3	3	4	4	1	19
FB6	1	3	3	3	2	3	1	16
FB7	1	3	3	3	2	3	1	16
FB8	3	3	3	3	3	3	1	19
FB13	1	2	3	1	5	1	1	14
FB14	3	3	3	3	3	3	1	19
FB15	3	3	3	2	3	3	1	18
FB16	2	2	3	3	4	3	1	18
FB17	3	2	3	3	4	3	1	19
FB19	1	1	1	1	1	1	1	7
FB20	2	2	2	2	3	2	1	14
FB12	2	2	2	2	3	3	1	15

Samples	Somatic concern	Anxiety	Guilt feelings	Tension	Mannerisms & posturing	Depression	Motor retardation	UncooperatJveness
FB1	1	5	1	5	1	1	1	6
FB2	3	4	3	3	1	5	3	2
FB3	1	1	1	1	1	1	4	4
FB4	2	5	2	5	1	5	4	3
FB5	1	4	1	4	1	2	1	6
FB6	1	4	1	4	1	1	1	6
FB7	1	3	1	4	1	1	1	5
FB8	1	4	1	4	1	1	1	6
FB13	1	6	1	5	1	2	1	6
FB14	1	5	1	4	1	1	1	6
FB15	1	3	1	3	1	1	1	5
FB16	1	4	1	4	1	1	1	6
FB17	1	4	1	4	1	1	1	5
FB19	1	3	1	3	1	1	1	4
FB20	1	4	1	3	1	1	1	5
FB12	1	4	1	3	1	1	1	6

Samples	Unusual thought content	Disorientation	Poor attention	Lack of judgment & insight	Disturbance of volition	Poor impulse control	Preoccupation	Active social avoidance	General Psychopathology Scale
FB1	2	1	5	6	3	5	1	4	48
FB2	1	1	4	5	5	3	1	4	48
FB3	3	1	5	6	5	2	1	6	43
FB4	1	1	4	4	4	2	1	5	49
FB5	1	1	5	6	1	6	1	4	45
FB6	1	1	5	5	1	6	1	3	42
FB7	1	1	4	5	1	5	1	4	39
FB8	1	1	5	6	2	6	1	5	46
FB13	3	1	7	7	1	7	1	5	55
FB14	2	1	5	5	4	6	1	4	48
FB15	1	1	5	5	4	4	1	3	40
FB16	4	1	5	6	1	6	1	4	47
FB17	3	1	5	6	2	6	1	4	46
FB19	3	1	4	5	1	5	1	3	38
FB20	3	1	4	5	2	5	1	3	41
FB12	3	1	2	6	3	6	1	3	43

Samples	visuospatial executive	naming	attention	calculation	language	abstraction	delayed recall	orientation	total MoCA

FB1	4	3	3	3	2	3	3	6	27
FB2	4	3	3	1	2	2	5	6	26
FB3	5	3	3	3	3	1	1	6	25
FB4	4	3	2	3	2	2	1	6	23
FB5	5	2	2	3	1	1	0	6	20
FB6	4	3	3	1	3	2	0	6	22
FB7	3	3	3	3	3	2	4	6	27
FB8	3	2	3	3	1	1	3	6	22
FB13	3	3	3	3	2	2	5	6	27
FB14	4	3	2	3	1	1	2	6	22
FB15	4	3	3	2	2	2	1	6	23
FB16	3	3	3	3	2	1	2	6	23
FB17	4	3	3	3	2	2	2	6	25
FB19	5	3	3	3	3	2	3	6	28
FB20	4	3	2	3	3	2	2	6	25
FB12	5	3	3	3	2	1	2	6	25

FB belong to the METH group, FBC belong to the Ctr group.
Lower scores indicate less severe symptoms.

Supplementary Table 6. Spearman rank correlation between the microbes and the scales of PNASS and MoCA tests.

positive scale

R	unidentified_Enterobacteriaceae	unidentified_Ruminococcaceae	Bacteroides	Bifidobacterium	Blautia	Rombo utsia	Collinsella	Faecalibacterium	unidentified_Lachnospiraceae	Streptococcus
Delusions	0.6162	-0.4885	0.5230	-0.3667	-0.0240	-0.4569	-0.1323	-0.2435	0.2450	-0.0240
Conceptual disorganization	0.0152	-0.0744	0.2131	-0.0406	0.1675	-0.0660	-0.1235	-0.3045	0.2774	0.1793
Hallucinatory behavior	0.3855	-0.1189	0.1240	-0.2241	0.0458	-0.1392	-0.0696	-0.0560	0.1511	0.3447
Excitement	0.3619	-0.3969	0.1718	-0.3543	0.0715	0.0791	-0.2281	-0.0547	0.1566	-0.0745
Grandiosity	0.3081	-0.3641	-0.3081	0.0840	0.4201	0.3081	-0.1960	-0.3641	0.3641	0.0840
Suspiciousness	0.5683	-0.4697	0.4482	-0.3157	-0.0370	-0.3958	-0.0662	-0.0801	0.1710	-0.1679
Hostility	0.2881	-0.3506	0.0412	-0.3597	0.1692	0.2317	-0.1753	-0.0290	0.1494	-0.2012
Positive Scale	0.6204	-0.5067	0.2836	-0.3811	0.1152	-0.1891	-0.2496	-0.2245	0.3117	0.1211

p value	unidentified_Enterobacteriaceae	unidentified_Ruminococcaceae	Bacteroides	Bifidobacterium	Blautia	Romboutsia	Collinsella	Faecalibacterium	unidentified_Lachnospiraceae	Streptococcus
Delusions	0.0110	0.0549	0.0376	0.1624	0.9296	0.0752	0.6253	0.3635	0.3605	0.9296
Conceptual disorganization	0.9554	0.7841	0.4281	0.8813	0.5353	0.8082	0.6487	0.2516	0.2983	0.5064
Hallucinatory behavior	0.1404	0.6611	0.6474	0.4040	0.8661	0.6070	0.7978	0.8367	0.5764	0.1910
Excitement	0.1684	0.1280	0.5246	0.1782	0.7925	0.7710	0.3955	0.8404	0.5624	0.7839
Grandiosity	0.2457	0.1657	0.2457	0.7571	0.1052	0.2457	0.4668	0.1657	0.1657	0.7571
Suspiciousness	0.0216	0.0664	0.0817	0.2336	0.8919	0.1291	0.8075	0.7681	0.5267	0.5343
Hostility	0.2793	0.1831	0.8797	0.1712	0.5311	0.3879	0.5161	0.9152	0.5809	0.4549
Positive Scale	0.0103	0.0452	0.2871	0.1453	0.6709	0.4831	0.3511	0.4032	0.2399	0.6550

The negative scale

R	unidentified_Enterobacteriaceae	unidentified_Ruminococcaceae	Bacteroides	Bifidobacterium	Blautia	Romboutsia	Collinsella	Faecalibacterium	unidentified_Lachnospiraceae	Streptococcus
Blunted affect	-0.1109	0.0832	-0.1386	0.5698	0.1725	-0.2495	0.0770	-0.3481	0.0647	0.3881
Emotional withdrawal	-0.4747	0.3518	-0.6192	0.5608	0.2658	0.3411	0.0046	-0.2151	0.0384	0.4148
Poor rapport	-0.3204	0.2452	-0.4367	0.5817	0.4493	0.2041	0.1002	-0.4511	0.3132	0.3490
Passive-apathetic social withdrawal	-0.4242	0.3213	-0.3767	0.6521	0.1551	0.1219	0.0032	-0.1361	0.0142	0.5682
Difficulty in abstract thinking	-0.0338	-0.0676	-0.0369	0.2212	0.3011	-0.3503	-0.2397	-0.5101	0.5009	0.5485
Lack of spontaneity & flow of conversation	-0.7143	0.5373	-0.2078	0.5942	-0.0179	-0.1120	0.0536	0.0260	-0.1753	0.5649
Stereotyped thinking	NA	NA	NA	NA	NA	NA	NA	NA	NA	NA
Negative Scale	-0.4529	0.2970	-0.3415	0.6504	0.1930	-0.0059	-0.0297	-0.2702	0.0564	0.6162

p value	unidentified_Enterobacteriaceae	unidentified_Ruminococcaceae	Bacteroides	Bifidobacterium	Blautia	Romboutsia	Collinsella	Faecalibacterium	unidentified_Lachnospiraceae	Streptococcus
Blunted affect	0.6827	0.7594	0.6087	0.0212	0.5229	0.3514	0.7768	0.1865	0.8119	0.1374

Emotional withdrawal	0.0631	0.1814	0.0105	0.0238	0.3197	0.1961	0.9865	0.4237	0.8877	0.1101
Poor rapport	0.2263	0.3600	0.0908	0.0181	0.0808	0.4484	0.7119	0.0795	0.2375	0.1852
Passive-apathetic social withdrawal	0.1015	0.2249	0.1504	0.0062	0.5662	0.6530	0.9907	0.6152	0.9582	0.0217
Difficulty in abstract thinking	0.9011	0.8036	0.8922	0.4103	0.2571	0.1835	0.3713	0.0435	0.0481	0.0278
Lack of spontaneity & flow of conversation	0.0019	0.0318	0.4400	0.0152	0.9477	0.6796	0.8438	0.9239	0.5160	0.0226
Stereotyped thinking	NA	NA	NA	NA	NA	NA	NA	NA	NA	NA
Negative Scale	0.0781	0.2640	0.1955	0.0064	0.4738	0.9826	0.9131	0.3114	0.8356	0.0110

the general psychopathology scale

R	unidentified_Enterobacteriaceae	unidentified_Ruminococcaceae	Bacteroides	Bifidobacterium	Blautia	Rombo utsia	Collinsella	Faecalibacterium	unidentified_Lachnospiraceae	Streptococcus

Somatic concern	-0.3017	0.2301	-0.3017	0.1892	-0.2096	-0.0205	-0.1508	-0.1074	-0.1815	0.4014
Anxiety	0.3890	-0.4713	-0.0269	-0.3732	-0.0127	-0.0127	-0.4855	-0.3384	0.1755	0.3384
Guilt feelings	-0.3017	0.2301	-0.3017	0.1892	-0.2096	-0.0205	-0.1508	-0.1074	-0.1815	0.4014
Tension	0.2380	-0.4321	-0.0282	-0.0219	0.0720	-0.0125	-0.3476	-0.2943	0.2004	0.1566
Mannerisms & posturing	NA	NA	NA	NA	NA	NA	NA	NA	NA	NA
Depression	-0.2440	0.0659	-0.0813	-0.0697	-0.0930	-0.1433	-0.3022	-0.1046	0.0775	0.3486
Motor retardation	-0.3803	0.3738	-0.3068	0.4624	0.0043	-0.0411	-0.0583	-0.2485	0.0540	0.5099
Uncooperat Jveness	0.3298	-0.3805	0.3298	-0.3266	-0.1459	-0.0539	-0.3155	0.0666	-0.0967	-0.0111
Unusual thought content	0.6057	-0.3827	0.4033	-0.3005	-0.0016	-0.3448	-0.1502	-0.2625	0.3084	0.1154
Disorientation	NA	NA	NA	NA	NA	NA	NA	NA	NA	NA
Poor attention	0.0891	-0.1155	0.0660	0.1023	0.3466	0.0495	0.0660	-0.4588	0.4522	-0.0132
Lack of judgment & insight	0.2832	-0.4023	0.4087	-0.0772	0.0290	-0.4344	-0.3958	-0.1513	0.2124	0.0869
Disturbance of volition	-0.2328	0.2359	-0.4246	0.3272	0.2009	0.0578	0.0609	-0.3394	0.0061	0.3972
Poor impulse control	0.3206	-0.3764	0.5220	-0.5452	-0.3005	-0.2122	-0.2540	0.1595	-0.1549	-0.1719
Preoccupation	NA	NA	NA	NA	NA	NA	NA	NA	NA	NA
Active social avoidance	-0.1065	-0.0031	-0.1597	0.4697	0.0595	-0.2035	-0.3789	-0.3162	0.1659	0.5730

General Psychopathology Scale	0.1861	-0.2821	-0.0812	-0.0266	-0.0414	-0.1654	-0.5037	-0.5022	0.1492	0.6174

p value	unidentified_Enterobacteriaceae	unidentified_Ruminococcaceae	Bacteroides	Bifidobacterium	Blautia	Rombo utsia	Collinsella	Faecalibacterium	unidentified_Lachnospiraceae	Streptococcus
Somatic concern	0.2562	0.3913	0.2562	0.4828	0.4358	0.9401	0.5771	0.6922	0.5011	0.1233

Anxiety	0.1364	0.0654	0.9213	0.1545	0.9629	0.9629	0.0566	0.1998	0.5155	0.1998
Guilt feelings	0.2562	0.3913	0.2562	0.4828	0.4358	0.9401	0.5771	0.6922	0.5011	0.1233
Tension	0.3748	0.0946	0.9175	0.9358	0.7910	0.9633	0.1872	0.2685	0.4568	0.5626
Mannerisms & posturing	NA	NA	NA	NA	NA	NA	NA	NA	NA	NA
Depression	0.3624	0.8085	0.7646	0.7975	0.7320	0.5964	0.2554	0.6999	0.7755	0.1857
Motor retardation	0.1462	0.1538	0.2477	0.0713	0.9873	0.8800	0.8301	0.3534	0.8425	0.0436
Uncooperat Jveness	0.2123	0.1460	0.2123	0.2169	0.5899	0.8428	0.2339	0.8064	0.7216	0.9675
Unusual thought content	0.0129	0.1435	0.1214	0.2581	0.9954	0.1910	0.5786	0.3260	0.2452	0.6703
Disorientation	NA	NA	NA	NA	NA	NA	NA	NA	NA	NA
Poor attention	0.7428	0.6701	0.8081	0.7061	0.1885	0.8555	0.8081	0.0739	0.0787	0.9613
Lack of judgment & insight	0.2879	0.1224	0.1160	0.7762	0.9152	0.0927	0.1291	0.5761	0.4297	0.7490
Disturbance of volition	0.3855	0.3791	0.1012	0.2161	0.4557	0.8315	0.8228	0.1985	0.9821	0.1277
Poor impulse control	0.2260	0.1508	0.0381	0.0290	0.2581	0.4301	0.3424	0.5551	0.5668	0.5243
Preoccupation	NA	NA	NA	NA	NA	NA	NA	NA	NA	NA
Active social avoidance	0.6948	0.9908	0.5547	0.0664	0.8268	0.4496	0.1479	0.2328	0.5391	0.0203

General Psychopathology Scale	0.4901	0.2897	0.7649	0.9221	0.8791	0.5403	0.0467	0.0474	0.5813	0.0108

MoCA

R	unidentified_Enterobacteriaceae	unidentified_Ruminococcaceae	Bacteroides	Bifidobacterium	Blautia	Romboutsia	Collinsella	Faecalibacterium	unidentified_Lachnospiraceae	Streptococcus

visuospatial executive	-0.1917	0.2109	0.2684	-0.1917	0.0192	-0.0192	0.0959	0.1342	0.0383	-0.0192
naming	0.2460	0.0820	-0.2870	-0.2050	0.2460	0.2460	0.3280	-0.4920	0.2460	-0.1230
attention	0.1566	-0.1566	0.1252	0.1566	-0.1252	-0.0626	0.0000	-0.2192	-0.1252	-0.4070
calculation	0.4127	-0.4559	0.2658	-0.0259	0.0238	-0.3889	-0.4473	0.0194	0.1880	0.3435
language	0.0032	0.2934	-0.2181	-0.0016	0.1876	0.3351	0.4169	0.0545	0.2197	-0.4313
abstraction	0.1423	-0.1339	-0.4435	-0.0921	0.5021	0.4184	0.2678	-0.3096	0.4603	-0.4770
delayed recall	0.3790	-0.2978	-0.2497	-0.2377	-0.0707	0.1248	-0.3460	-0.0692	-0.1173	-0.0391
orientation	NA	NA	NA	NA	NA	NA	NA	NA	NA	NA
total MoCA	0.3722	-0.2078	-0.2183	-0.2257	0.2452	0.1495	-0.0688	-0.2467	0.2706	-0.2541

p value	unidentified_Enterobacteriaceae	unidentified_Ruminococcaceae	Bacteroides	Bifidobacterium	Blautia	Rombotsia	Collinsella	Faecalibacterium	unidentified_Lachnospiraceae	Streptococcus
visuospatial executive	0.4768	0.4330	0.3148	0.4768	0.9438	0.9438	0.7239	0.6202	0.8879	0.9438

naming	0.3585	0.7627	0.2812	0.4463	0.3585	0.3585	0.2149	0.0529	0.3585	0.6500
attention	0.5626	0.5626	0.6440	0.5626	0.6440	0.8178	1.0000	0.4147	0.6440	0.1176
calculation	0.1121	0.0759	0.3198	0.9241	0.9304	0.1365	0.0824	0.9430	0.4857	0.1926
language	0.9906	0.2700	0.4172	0.9953	0.4866	0.2045	0.1082	0.8411	0.4137	0.0953
abstraction	0.5992	0.6211	0.0853	0.7346	0.0475	0.1068	0.3160	0.2432	0.0728	0.0617
delayed recall	0.1477	0.2626	0.3510	0.3755	0.7947	0.6450	0.1893	0.7990	0.6652	0.8857
orientation	NA	NA	NA	NA	NA	NA	NA	NA	NA	NA
total MoCA	0.1557	0.4400	0.4167	0.4005	0.3601	0.5805	0.8002	0.3571	0.3108	0.3422

correlation coefficient -1<R<1, R >0 positve correlation, R<0 negative correlation.
