# Supplementary figures and images for: Altered fecal microbiota composition in individuals who abuse methamphetamine
Source: Sci Rep. 2021 Sep 13;11:18178. doi: 10.1038/s41598-021-97548-1 (PMC8437956; doi:10.1038/s41598-021-97548-1)

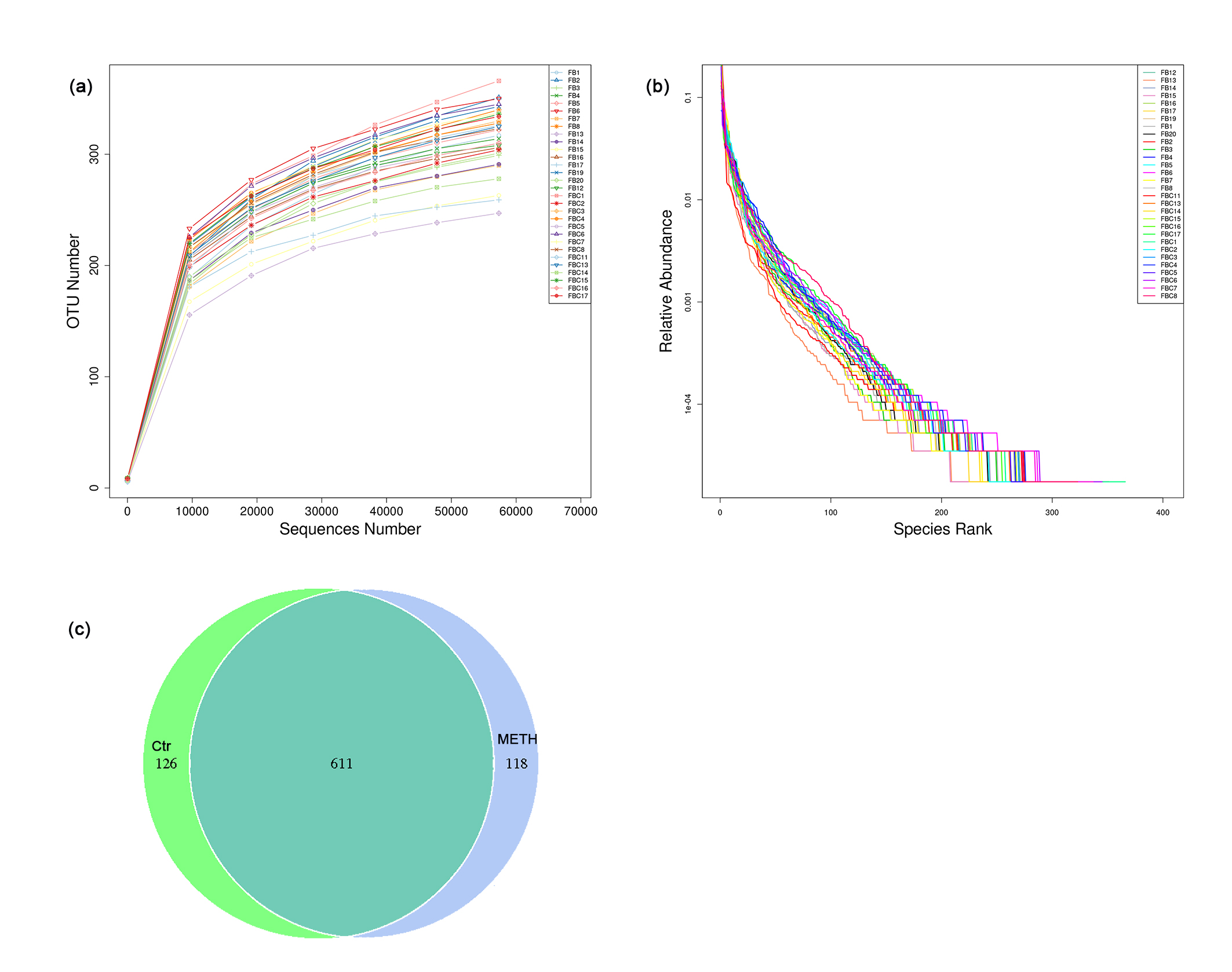

Supplement: Supplementary file 2 — Supplementary Figure 1. [file 41598_2021_97548_MOESM2_ESM.jpg]

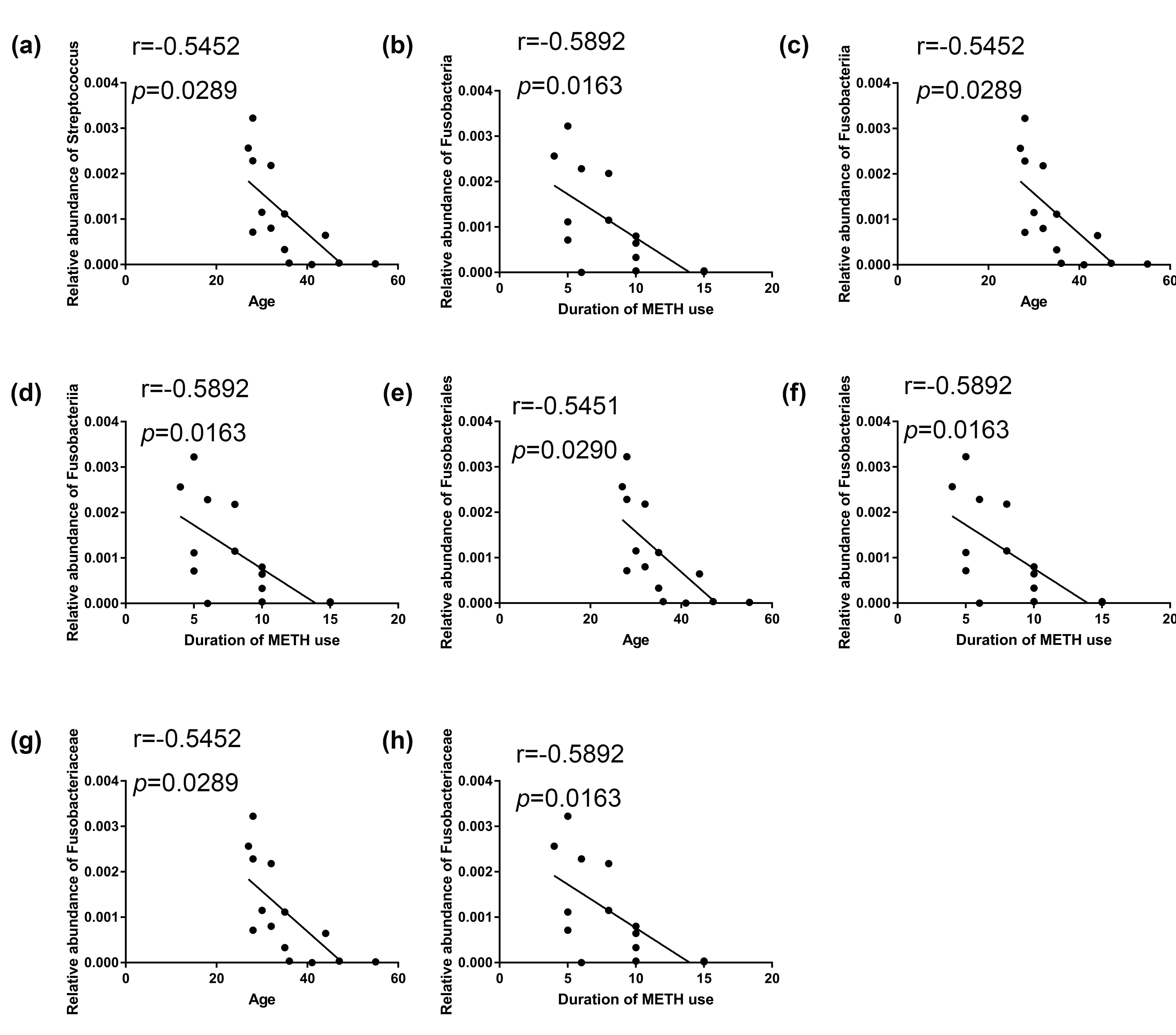

Supplement: Supplementary file 3 — Supplementary Figure 2. [file 41598_2021_97548_MOESM3_ESM.jpg]

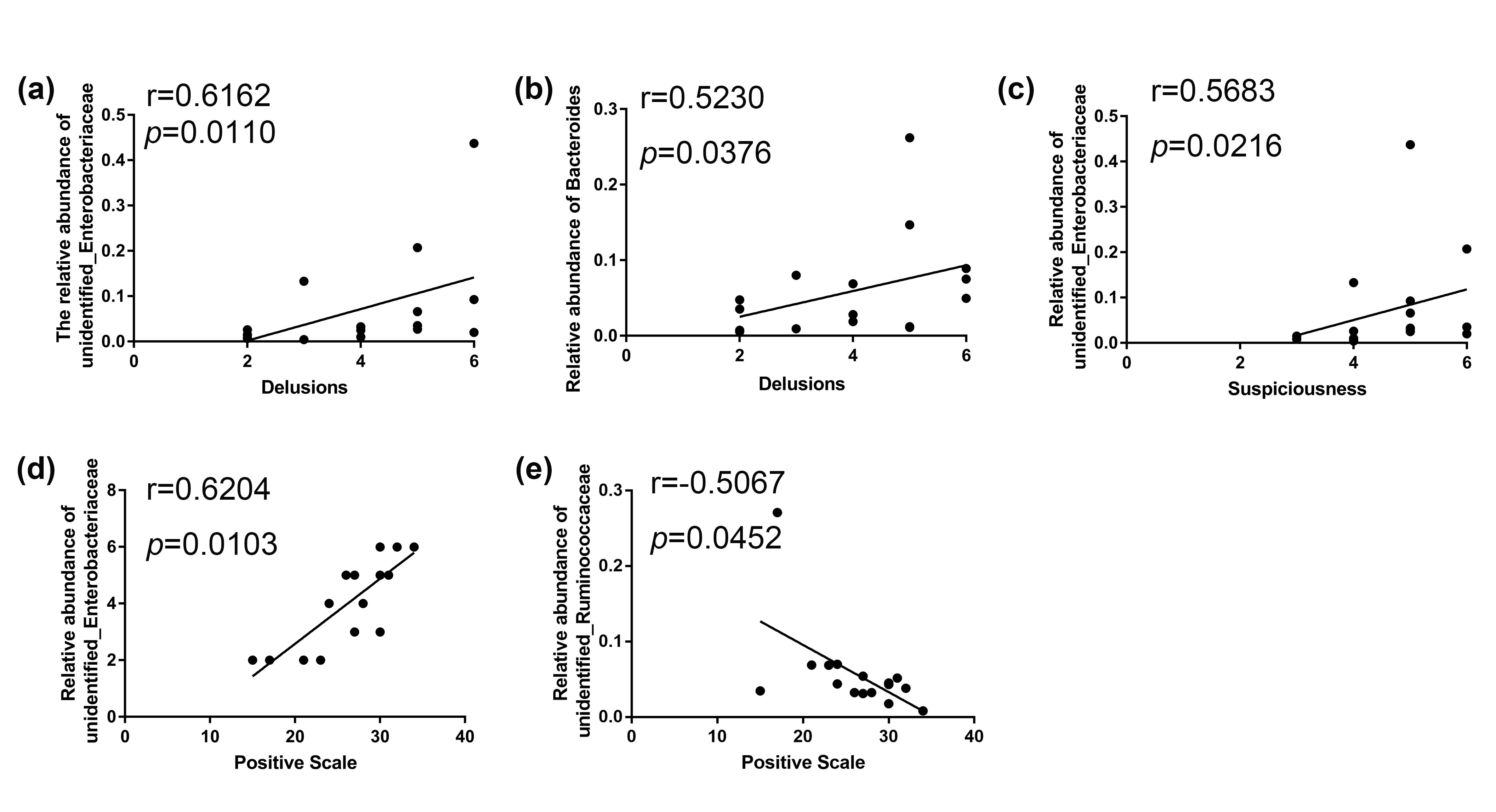

Supplement: Supplementary file 4 — Supplementary Figure 3. [file 41598_2021_97548_MOESM4_ESM.jpg]

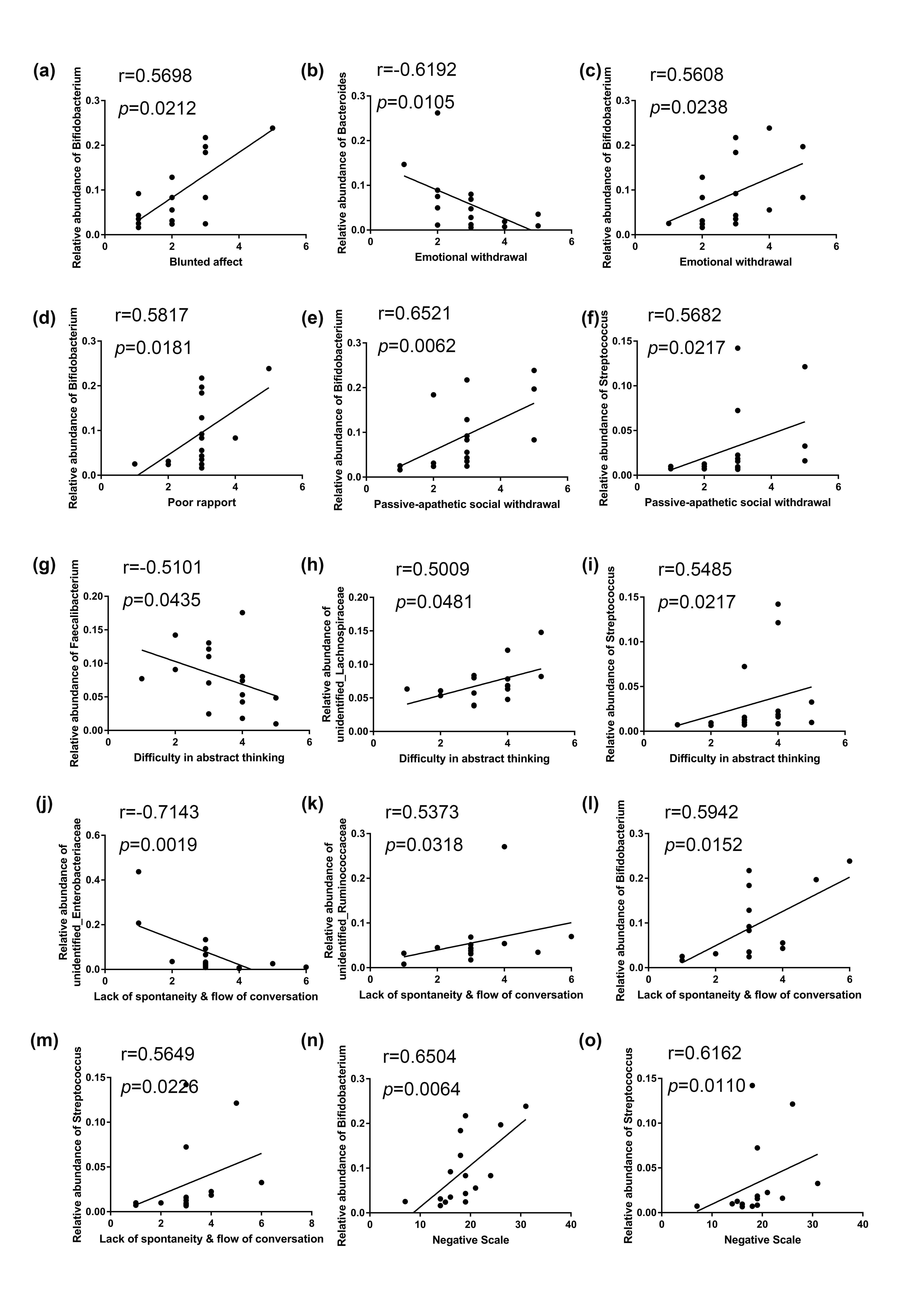

Supplement: Supplementary file 5 — Supplementary Figure 4. [file 41598_2021_97548_MOESM5_ESM.jpg]

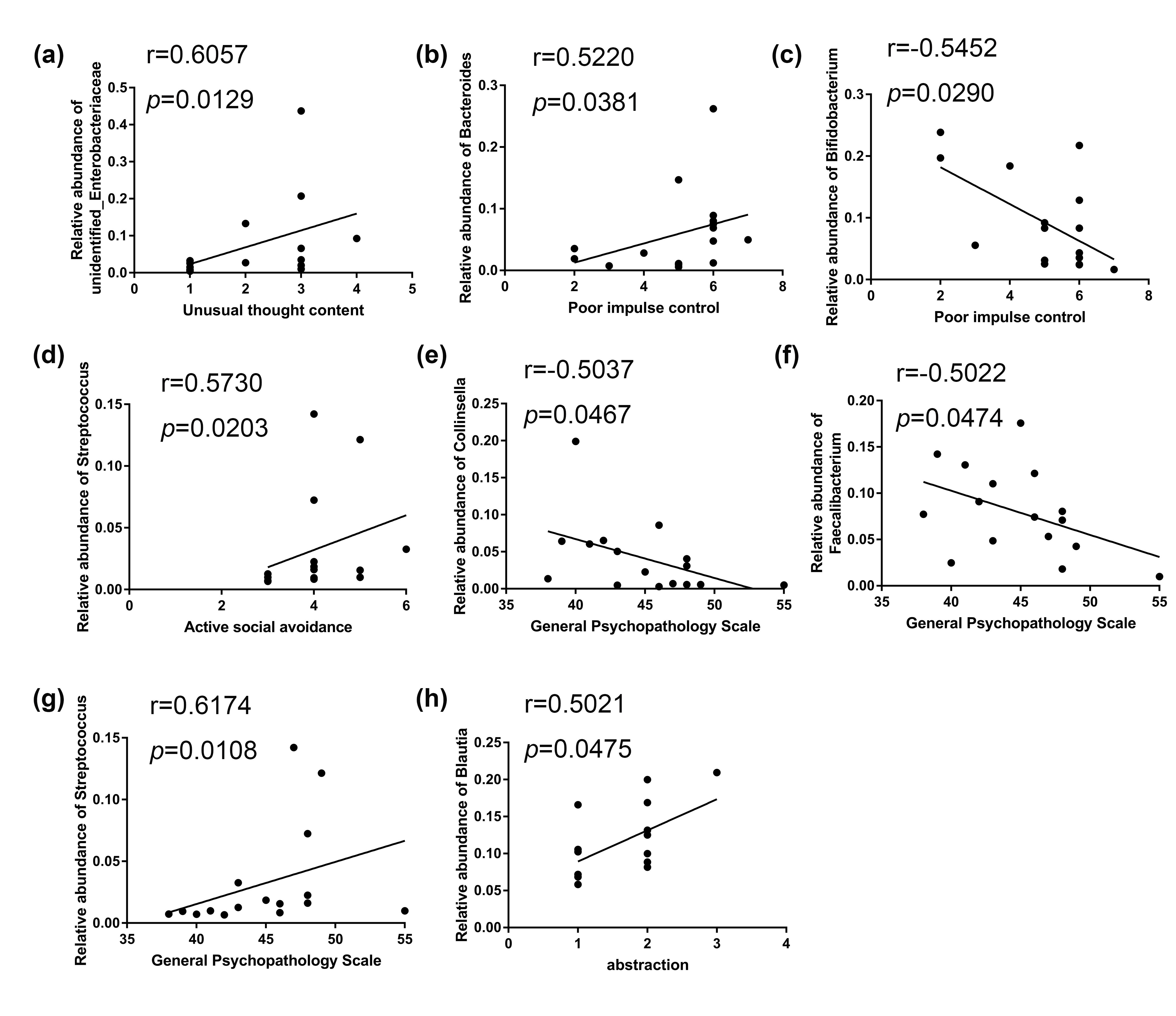

Supplement: Supplementary file 6 — Supplementary Figure 5. [file 41598_2021_97548_MOESM6_ESM.jpg]
